# High Entropy Layered Cathode With Single Grain Morphology for High‐Performance Sodium‐Ion Batteries

**DOI:** 10.1002/smll.202511833

**Published:** 2026-01-15

**Authors:** Daniele Callegari, Giulia Maranini, Claudia Triolo, Mariam Maisuradze, Hemanth Kumar Beere, Abdelhaq Nassiri, Umberto Anselmi‐Tamburini, Saveria Santangelo, Marco Giorgetti, Mauro Coduri

**Affiliations:** ^1^ Department of Chemistry University of Pavia Pavia Italy; ^2^ INSTM Firenze Italy; ^3^ Department of Civil Energy, Environmental and Materials Engineering (DICEAM) Mediterranean University Reggio Calabria Italy; ^4^ National Reference Center for Electrochemical Energy Storage (GISEL) Firenze Italy; ^5^ Department of Industrial Chemistry “Toso Montanari” University of Bologna Bologna Italy

**Keywords:** high entropy oxide, layered cathode materials, sodium ion batteries, spray‐pyrolysis

## Abstract

A major obstacle in the advancement of sodium‐ion batteries (SIBs) is the development of cathode active materials (CAMs) that offer both high specific capacity and long‐term cycling stability. Among the various candidates, layered CAMs have attracted significant attention. In this work, we synthesized a high‐entropy layered CAM with composition (Na_0.52_Ti_0.19_Mn_0.19_Fe_0.21_Ni_0.21_Co_0.20_O_2_) using a spray pyrolysis technique, yielding large (0.75 µm on average) and separated grains. The resulting material comprises a P3–O3 layered oxide mixture, along with ∼20% rock‐salt and spinel phases. This CAM demonstrates a high specific capacity (∼180 mAh g^−1^ at 0.08 C), excellent rate capability (69% retention after 300 cycles at 1C), and high coulombic efficiency (>99.5%). In comparison, a CAM of identical composition synthesized via a conventional sol–gel method, exhibiting an agglomerated microstructure, showed lower capacity and retention, consistent with literature reports. These findings highlight the advantages of combining high entropy design and cathode morphology in developing next‐generation cathodes for SIBs.

## Graphical abstract

We produced a multiphase high entropy layered cathode with nominal composition Na_0.52_Ti_0.19_Mn_0.19_Fe_0.21_Ni_0.21_Co_0.20_O_2_ and single grain morphology, which demonstrated enhanced specific capacity (∼180 mAh g^−1^ at 0.08 C), excellent rate capability (69% retention after 300 cycles at 1C), and high coulombic efficiency (>99.5%).

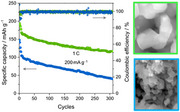

## 1 Introduction

Sodium‐ion batteries (SIBs) have emerged as a promising alternative to traditional lithium‐ion batteries (LIBs), owing to their sustainability, resource abundance, and superior safety features [1, 2, 3]. Unlike LIBs, which rely on a critical material such as lithium, SIBs utilize sodium, an element abundantly available in the Earth's crust and seawater, aligning with global efforts for sustainable energy storage solutions [4]. Yet, SIBs face significant challenges in terms of energy density and cycle life, which must be addressed for them to be competitive in applications such as electric vehicles and large‐scale energy storage systems [5].

A critical aspect in the development of SIBs is the production of stable and high‐performing cathodes [6]. Various chemistries have been explored to enhance their capacity, structural stability, and long‐term performance [7, 8, 9]. Among the most promising candidates are layered oxides (NaTMO_2_, where TM = transition metal), which feature a well‐ordered arrangement of Na^+^ layers that enable efficient sodium insertion and extraction, enhancing both capacity and stability during charge–discharge cycles. Layered phases are named after Delmas notation [10], with P and O standing for prismatic or octahedral coordination of the TM ion, followed by a number indicating the number of intermediate layers. Each polymorph has characteristic interlayer distances and local chemical environments, which impact the Na insertion/release process and contribute to specific electrochemical properties. The polymorph type depends on the Na content and on the nature of the TM cation. Fully sodiated compounds typically adopt a rhombohedral O3 structure [11, 12, 13], or a monoclinic distortion [14, 15], with limited specific capacities (90–120 mAh g^−1^) but excellent long‐term stability [16, 17]. The P polymorphs, instead, offer larger capacities (up to 160 mAh g^−1^ during initial cycles), which degrade significantly over time due to irreversible transformations during charge/discharge cycles [18]. Overall, each polymorph has its trade‐offs in terms of energy capacity, ionic conductivity, and cycling stability. Selecting the appropriate cathode depends on the specific application requirements. Often, a mixture of different layered phases is preferred to achieve a well‐rounded performance [19].

An emerging direction in cathode research for SIBs involves the exploration of high‐entropy oxides (HEOs), where multiple cations occupy a single crystallographic site, leading to properties that may differ from those of the individual components [20]. The disordered atomic arrangements of HEOs enhance thermal and mechanical stability, minimizing structural degradation and boosting the battery's lifespan and reliability [19, 21]. The structural disorder also enhances Na^+^ diffusion, increasing ionic and electrical conductivity, both crucial for operating high‐performance SIBs. Recent studies showed that HEO cathodes can achieve initial specific capacities between 150‐180 mAh g^−1^, maintaining substantial capacity retention after 300 cycles [22, 23, 24]. O3‐type (NaNi_0.12_Cu_0.12_Mg_0.12_Fe_0.15_Co_0.15_Mn_0.1_Ti_0.1_Sn_0.1_Sb_0.04_O_2_) exhibits remarkable cycling stability (∼83% retention after 500 cycles) and rate performance (∼80% of capacity retention at 5.0C) [21]. Its reversible O3−P3 phase transition and the entropy‐stabilized multi‐cation matrix effectively suppress structural degradation and accommodates local distortions during Na^+^ (de)intercalation. This combination of high capacity and long lifespan positions HEOs as a promising direction for the future of SIBs. Ding et al. [25] revealed that precise cation substitution in layered O3‐type Na cathodes critically governs structural stability and electrochemical performance. In NaNi_0.3_Cu_0.1_Fe_0.2_Mn_0.3_M_0.1_O_2_ (M = Ti, Sn), replacing Sn^4+^ (0.69 Å) with smaller Ti^4+^ (0.61 Å) reduces lattice strain (ε ≈ 0.01%) and improves atomic coordination uniformity. The Ti‐based phase (NaNi_0.3_Cu_0.1_Fe_0.2_Mn_0.3_Ti_0.1_O_2_) thus exhibits enhanced mechanochemical compatibility, faster Na^+^ diffusion, and superior redox reversibility, whereas Sn substitution induces planar strain, lattice distortion, and capacity fading. Beyond the chemical composition, factors such as particle size and morphology, porosity, and crystallinity directly influence ionic conductivity, structural integrity, and charge/discharge efficiency [18]. Materials with large, defect‐free grains (often referred to as “single‐crystal” cathodes, SCCs) are less prone to forming microcracks or voids, which can result from volume changes during cycling and lead to capacity loss [26, 27].

Inspired by the potential of SCCs, we exploited a spray pyrolysis (SP) method (details in Supporting Information) to synthesize a layered high‐entropy cathode active material (SP‐CAM) with controlled morphology. This SP‐CAM consists of an equimolar mixture of five TMs (Ti, Mn, Fe, Co, Ni) and a limited fraction of Na (∼0.5−0.6 per formula unit) to promote the formation of a multi‐phase layered system. In the absence of oxygen non‐stoichiometry, the configurational entropy (*S_conf_*) can be estimated for both O3 and P3 polymorphs as [20]:

(1) $$S_{conf}=-R\left(\sum_{i,Na}\chi_{i}\ln\chi_{i}+\sum_{i,TM}\chi_{i}\ln\chi_{i}\right)$$

where R is the universal gas constant, and χ_*i*_ denotes the molar fraction of each species sharing a given crystallographic site, in this case those related to Na and TM ions. When the Na site is half‐filled, *S_conf_* amounts to 2.30 R, thus exceeding 1.5 R, a commonly cited heuristic threshold for high‐entropy oxides [28].

The TMs were chosen to provide charge compensation during the (de)sodiation process, mitigating the structural distortions that can hamper the stability of the cathode. To examine the effect of the microstructure, we produced another CAM with very similar composition following a conventional sol–gel procedure (SG), with final annealing at 700°C. The powders and the corresponding fresh and used electrodes were characterized by combining X‐ray diffraction (XRD), scanning electron microscopy (SEM), micro‐Raman spectroscopy (μRS), and X‐ray absorption spectroscopy (XAS). Despite the same chemical composition, the SP cathode (SPC) exhibited impressive specific capacity and retention after 300 cycles. The different behaviour is associated with the peculiar microstructure and the different elements of the active layered phases.

## 2 Results and Discussion

**FIGURE 1:**
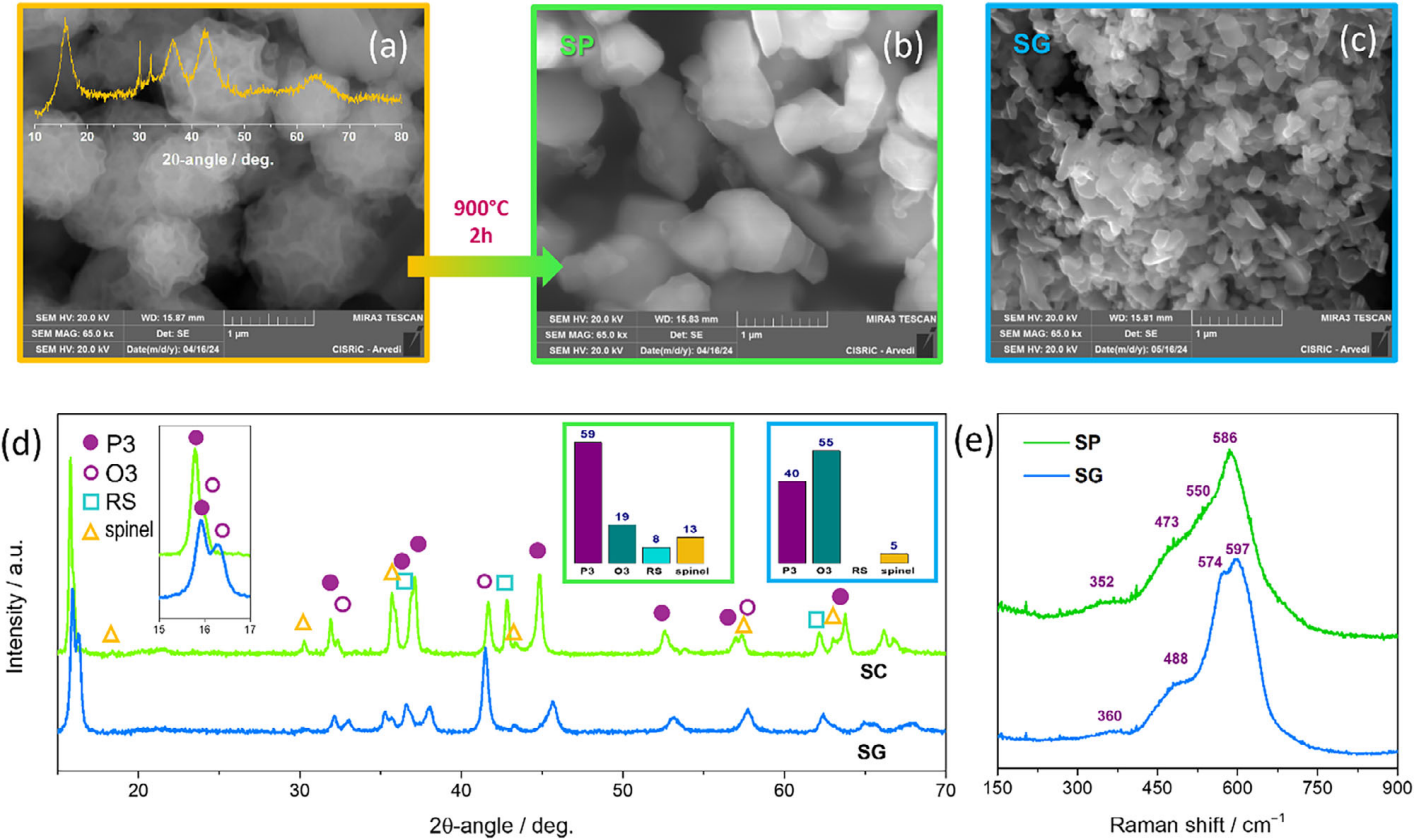
Morphological and structural characterization of SP‐ and SG‐CAMs. (a) SEM and XRD of the SP‐CAM precursor from spray pyrolysis; (b,c) SEM images with secondary electrons; (d) XRD with phase fractions in histogram; (e) Raman spectra.

The elemental composition of the SP‐CAM, determined using inductively coupled plasma (ICP) for Na and SEM‐EDS (energy dispersion spectroscopy) for TMs, is Na_0.52_Ti_0.19_Mn_0.19_Fe_0.21_Ni_0.21_Co_0.20_O_2_ (see Supporting Information). The powders produced via pyrolysis at 900°C displayed a cauliflower‐like morphology with submicron dimensions (Figure 1a). The corresponding XRD pattern revealed a large fraction of barely crystalline rock‐salt (RS), which is the native phase of NiO and CoO, along with a layered oxide with O3 structure. A rough estimate using the Scherrer formula indicated a crystallite size of ∼3 nm, suggesting that each particle consists of smaller crystals. The few sharp diffraction signals indicated the presence of residual NaNO_2_ (Supporting Information). To promote the formation of layered compounds, the powders were further annealed for 2 h at 900°C, followed by rapid cooling. As shown in Figure 1b, this resulted in larger, more distinct particles with submicron‐scale sizes (0.75 µm, on average). XRD evidenced a mixture of P3 (59.5%) and O3 (19.3%) layered polymorphs (Figure 1d), together with ∼8% spinel and ∼13% RS phases, which can be attributed to ∼Ni_0.5_Co_0.5_O and Fe_3_O_4_, respectively, based on the lattice parameters [29, 30]. Such a large content of RS likely derives from the same phase nucleated during the initial pyrolysis. As Na^+^ ions do not intercalate into RS and spinel, and no other phases were observed, Na likely accumulates in the active layer phases. The composition of the active layered phases, poor in Fe, Co, and Ni for the presence of spinel and RS, can be estimated as Na_0.80_(Ni_0.17_Co_0.13_Fe_0.11_Mn_0.29_Ti_0.29_)O_2_ (see Supporting Information for details). It follows that the corresponding value of *S_conf_* lowers from 2.30 to 2.03 R. More importantly, as discussed in the Supporting Information, this may still not reflect the true configurational entropy, which depends on the exact distribution of cations between the two layered phases.

As shown in Figure 1c, the SG‐CAM is composed of an irregular matrix of needle‐like particles, with smaller average particle dimensions (0.30 µm, on average). It showed less impurities (∼5% of spinel) and a prominence of O3 (55%) compared to P3 (40%) polymorphs (Figure 1d). The lattice parameters of the layered compounds varied significantly between the two cathodes (Supporting Information), consistently with a different distribution of Na and TM cations across the layered phases. Both CAMs showed a contraction in the *c*‐axis and expansion in the *a*‐axis when transitioning from the P3 to O3 polymorph, suggesting, as expected, a higher Na content in the latter phase.

The spatial homogeneity of the CAMs was evaluated by μRS. The lack of shifts and changes in relative intensities of the bands recorded at different random positions in each specimen (Supporting Information) indicates that both CAMs are spatially homogeneous. The average spectra are displayed in Figure 1e. They exhibit the characteristic spectrum of layered oxides [31, 32], which are similar for different polymorphs. The most intense band arises from the *A*_1g_ symmetry M─O stretching mode [31, 32], whereas the weakest contribution at 352−360 cm^−1^ originates from the *E*_g_ symmetry stretching vibration of the Na─O bonds [33]. The band at 473−488 cm^−1^ corresponds to the *E*_g_ symmetry O─M─O bending mode [31, 32]. Minor differences between the spectra of the CAMs are attributed to variations in the layered phases composition, in keeping with XRD. The *A*_1g_ mode (in O3 at lower frequencies than in P3 [31, 32]) is unresolved in the SP‐CAM (apparent peak position: ∼590 cm^−1^), whereas in the SG‐CAM it splits into two components owing to the larger O3 fraction. The shoulder on the higher‐frequency tail of the main O3/P3 *A*_1g_ mode of both CAMs originates from the *A*_1g_ mode [34, 35] of spinel impurities, and it is weaker in the SP‐CAM. In the latter, the one‐phonon longitudinal‐optical mode from defective RS [36] impurities contributes to the intensity at 550 cm^−1^.

**FIGURE 2:**
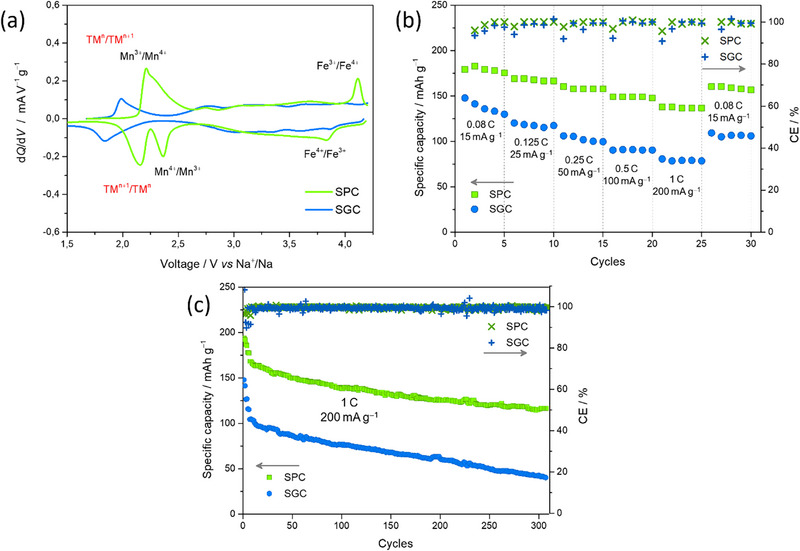
Electrochemical characterization of SPC and SGC. (a) 2^nd^ cycle of the dQ/dV curve performed between 1.5 and 4.2 V at 0.08C. (b) Comparison between discharge capacity, coulombic efficiency CE and rate capability test at different current densities. (c) Long‐term cycling performed for 300 cycles at 1C (200 mA g^−1^ from 4.2 to 1.5 V).

The electrochemical activity of the cathodes was ascertained by Potentiodynamic Cycling with Galvanostatic Acceleration (PCGA) with a C‐rate of 0.08C between 1.5 and 4.2 V. To mitigate electrolyte degradation at higher potentials (>4 V) during cycling, 2 wt.% FEC was added to the electrolyte [37, 38]. Figure 2a shows the dQ/dV curves as a function of voltage (Na^+^/Na) for the second cycle of the SPC and SG cathode (SGC). While the curves are qualitatively similar, the SGC shows more blurred signals, likely due to differences in redox kinetics and variations in the TMs stoichiometry. A comparison with existing data on layered compounds suggests that the peak around 2.5 V is consistent with the Mn^3^^+^/Mn^4+^ redox couple, while the peak near 2.0 V may correspond to redox activity of one of the other TMs [39, 40, 41, 42]. Given the complexity of the high‐entropy system, we cannot exclude that even the same Mn couple operates at different potentials across various layered polymorphs. The symmetry of the SPC peaks indicates that the reaction is highly reversible, which is promising for the long‐term cycling stability of the cathode. A peak at higher voltages (∼4.0 V), much more clearly defined for the SPC, is attributed to the Fe^3^^+^/Fe^4+^ couple [43]. This difference may indicate a more active electrochemical contribution of Fe in the SPC, potentially due to variations in crystal structure, Fe distribution between O3/P3 polymorphs, or enhanced electronic conductivity. These observations point to a strong dependence on the microstructure and the stoichiometric balance of the layered phases.

The electrochemical performance was further evaluated through a rate capability test. The SGC exhibited good performance and stability across all C‐rates (Figure 2b), consistent with previous reports. In contrast, the SPC showed a significant improvement in performance and stability, delivering an initial discharge capacity of ∼180 mAh g^−1^ at 0.08C (15 mA g^−1^), and maintaining over 130 mAh g^−1^ even at a high current density of 200 mA g^−1^ (1C). Across all C‐rates, the SPC consistently outperformed the SGC by ∼60 mAh g^−1^ during both charge and discharge cycles.

The long‐term stability test was performed at 1C rate up to 300 cycles. At the end of the test, the SPC retained a discharge capacity of about 120 mAh g^−1^, corresponding to 69% capacity retention, indicating its ability to endure numerous cycles at high current densities with minimal stability loss. Additionally, the coulombic efficiency (Figure 2c) remained above 99.5% throughout the entire process. Conversely, the SGC showed a considerable performance drop during the initial cycles at moderate current densities, achieving only 35% capacity retention by the end of the test, with a coulombic efficiency around 99.2%. The performance of SPC is competitive with benchmark layered oxides available in the literature (see Supporting Information for comparisons). For instance, Na(Ni_0.3_Fe_0.2_Mn_0.5_)_0.85_Ti_0.10_Co_0.05_O_2_ retains 78% of its capacity over 300 cycles with a similar initial discharge capacity (174.7 mAh g^−1^), while NaMn_0.2_Fe_0.2_Co_0.2_Ni_0.2_Sn_0.1_Al_0.05_Mg_0.05_O_2_ shows only 60% retention [44]. Moreover, the high‐rate performance of SPC (∼138 mAh g^−1^ at 1C) is comparable to that of Na(MgCu)_1/12_(NiCoFeMnTi)_1/6_O_2_, which delivers a discharge capacity of 128.3 mAh g^−1^ at 100 mA g^−1^ after extended cycling [45].

It could be surprising to associate such an impressive cycling stability to a CAM with ∼20% of secondary phases, which are not supposed to play a direct role in Na^+^ uptake or release. However, recent studies have shown that the formation of a surface spinel layer [46] or a rock‐salt–type layer [47] on top of a layered structure can, in fact, serve as a protective function, e.g., by suppressing Mn disproportionation triggered by surface reactions, thus mitigating Mn dissolution and the associated capacity fading, ultimately enhancing the cycling stability of the CAM.

In contrast, SGC exhibits significantly lower stability, indicating poor structural robustness upon prolonged cycling. Overall, the SPC composition and morphology demonstrate a promising balance between capacity, rate capability, and cycling stability, positioning it among the most competitive layered oxide cathodes for sodium‐ion batteries reported to date.

**FIGURE 3:**
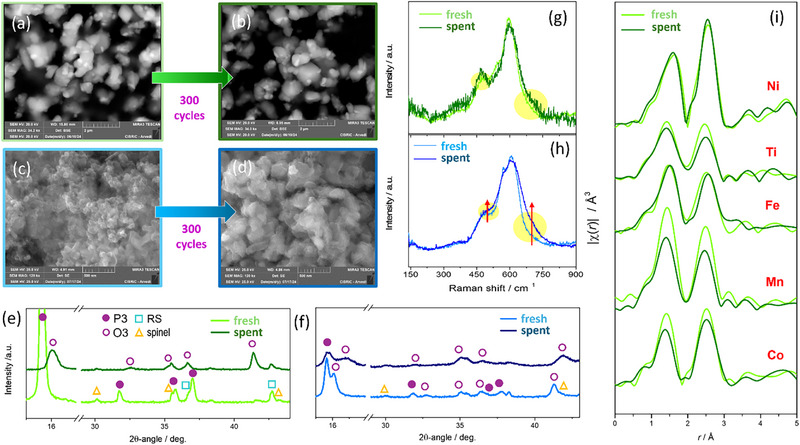
Characterization of SPC (green) and SGC (blue) in fresh (light) and spent (dark) conditions. (a‐d) SEM images with secondary electrons; (e,f) XRD patterns on selected angular regions; (g,h) Raman spectra; (i) FT‐EXAFS of SPC.

Figure 3a,d presents SEM images of the cathodes before and after electrochemical cycling. The SPC is characterized by well‐separated grains with submicrometric dimensions. No noticeable changes are observed after cycling. Similarly, XRD of the spent electrode reveals well‐crystalline material with a secondary phase content similar to that of the original powder (Figure 3e), while the layered polymorph is fully O3 due to different Na content at the end of the process. Lattice parameters and phase fractions are provided in the Supporting Information. No significant changes are observed in either the μRS spectra (Figure 3g,h), except for a noisier signal of the layered modes (<1000 cm^−1^) likely due to the low amount of material. Spectra collected from different regions confirmed the chemical homogeneity of the cathodes (see Supporting Information). The SGC is instead characterized by a complex matrix of interconnected grains, which increase in size after the long‐term cycling (Figure 3c,d). This change is not limited to grain morphology: XRD reveals a general loss of crystallinity, affecting preferentially the O3 phase (Figure 3f), whose short *c*‐axis (∼15.5 Å, see Supporting Information) suggests a very large content of Na or enrichment in small cations. This effect is even more evident after a few cycles at 200 mA g^−1^ (Supporting Information). Though spinel signals are apparent, a reliable phase quantification is prevented by the blurred O3 signals and peak overlaps. The growth of spinel was confirmed by μRS spectra, owing to the higher‐frequency tail of the M─O stretching mode of the P3/O3‐structure gathered from the spent electrode (Figure 3f) [35].

To investigate the chemical species responsible for the cathodes’ stability, XAS spectra were collected from both pristine and cycled electrodes (Figure 3i; Supporting Information). While no significant changes in the oxidation states were observed after cycling (Fe^3+^, Ti^4+^, Ni^2+^, Mn^4+^, Co^2+/3+^), as indicated by the XANES analysis (Supporting Information), the chemical environment around some ions changed in a different manner, despite all cations in each layered polymorph share the same crystallographic site should exhibit exactly the same environment, as confirmed by the FT‐EXAFS behaviors of the Figure 3i, in which all metals are characterized a similar local atomic environment with two main peaks, albeit with variations in intensity and/or distribution. Concerning SPC, Fe exhibited changes in the bond length of its first two neighbors (Fe─O and Fe─TM pairs), while Ti, Co and especially Mn underwent significant structural disorder, evidenced from the symmetric broadening of the interatomic distance distribution. Concerning Ti, the FT peak positions remain essentially unchanged, while only the intensity decreases in the spent electrode, therefore Ti experiences only moderate structural disorder. In this respect, Ti can act as a structural stabilizer without undergoing changes in its local environment. Surprisingly, the chemical environment of Ni remained unaltered. This behavior is independent from the Ni‐rich RS impurity, since the SGC behaved similarly (Supporting Information). In contrast, the SGC experienced more pronounced changes while pristine and cycled electrodes are compared, including greater disorder around Mn and especially Co ions. This may be due to the formation of a significant fraction of spinel phase and to the fact that the O3 phase of the SG‐CAM may intrinsically involve greater local structural deformation around these two metal centers. Therefore, the distinct behaviors of the two electrodes seem to be linked to different structural distortions around Fe, Ti, and Co ions, while Mn and Ni behave the same in both cathodes.

## 3 Conclusions

We have developed a high‐entropy cathode for SIBs with large oxide grains (0.75 µm, on average) that shows superior electrochemical performance, including high specific capacity, excellent rate capability, and coulombic efficiency. The stability of the SPC is confirmed by the minimal changes observed by XRD and μRS after cycling, which can be attributed to its peculiar morphology. Well‐separated grains are known to improve mechanical strength and structural stability during cycling, and the presence of the O3 polymorph, though a minority phase, may also contribute to its enhanced stability. The cathode contains a large fraction of RS and spinel phases, which may serve a protecting function enhancing the stability of the CAM. Additionally, the accumulation of RS and spinel increases the Na content in the layered phase to approximately 0.80 per formula unit (∼0.73 for P3, see Supporting Information for details). Interestingly, the SPC contains a higher proportion of the P3 phase, which is generally linked to limited cycling stability. In this case, however, several factors likely mitigate this effect: (i) its coexistence with the O3 polymorph, known for strong cycling stability, representing one‐third of the total layered content; (ii) the high‐entropy design of the overall composition; and (iii) the presence of large, well‐separated grains. Finally, the actual stoichiometry of the active phases, richer in Mn and Ti and poorer in Fe and Co, may further influence the electrochemical behavior of the system. One might argue that the larger capacity of the SPC compared to the SGC could be caused simply by the higher Na‐content in the layered phases. To test this, we produced another SG‐CAM with an actual Na content of 0.9 per formula unit. The resulting specific capacity recorded in a rate performance test was limited to ∼115 mAh g^−1^ (0.08C) and ∼70 mAh g^−1^ (1C) (see Supporting Information). Hence, a large Na content alone is not enough to achieve such high performance. Thus, we attribute the SPC's superior capacity to the unique chemical composition of the layered phases and larger oxide particles.

In this regard, XAS data revealed distinct local coordination for all cations and different structural evolutions after cycling. While the coordination around Ni remains fully reversible, the other transition metals experience varying degrees of disorder during cycling, reinforcing the idea that the exceptional performance is strongly tied to the specific chemical composition of the layered phases.

## 4 Experimental Section

Experiment details are given in the Supporting Information.

## Conflicts of Interest

The authors declare no conflicts of interest

## Supporting information




**Supporting file**: smll72218‐sup‐0001‐SuppMat.docx

## Data Availability

The data that support the findings of this study are available from the corresponding author upon reasonable request.
