# Supplementary material for: High Entropy Layered Cathode With Single Grain Morphology for High‐Performance Sodium‐Ion Batteries
Source: Small. 2026 Jan 15;22(11):e11833. doi: 10.1002/smll.202511833 (PMC12921545; doi:10.1002/smll.202511833)
Supplement: Supplementary file 1 — Supporting file: smll72218‐sup‐0001‐SuppMat.docx [file SMLL-22-e11833-s001.docx]

**Supporting Information**

**High Entropy Layered Cathode with Single-Grain Morphology for High-Performance Sodium-Ion Batteries**

Daniele Callegari^1,2^, Giulia Maranini^1^, Claudia Triolo^3,2,4^, Mariam Maisuradze^5^, Hemanth Kumar Beere^3^, Abdelhaq Nassiri^5^, Umberto Anselmi Tamburini^1,2^, Saveria Santangelo^3,2,4^*, Marco Giorgetti^5,2,4^*, Mauro Coduri^1,2^*

^1^Department of Chemistry, University of Pavia, Via Taramelli 16, 27100 Pavia, Italy

^2^INSTM, via G. Giusti 9, Firenze 50121, Italy

^3^Department of Civil, Energy, Environmental and Materials Engineering (DICEAM), Mediterranean University, Via Zehender, Loc. Feo di Vito, 89122 Reggio Calabria, Italy

^4^National Reference Center for Electrochemical Energy Storage (GISEL), via G. Giusti 9, Firenze 50121, Italy

^5^Department of Industrial Chemistry “Toso Montanari”, University of Bologna, Campus Navile, Via Piero Gobetti 85, 40139, Bologna, Italy

The supporting information reports the experimental details for the preparation of the powdered CAMs and the corresponding cathodes; a discussion about the estimate of the configurational entropy; the description of the experimental techniques used for the characterization of powders and cathodes; and details about the results of μRS, XRD, XAS and electrochemical testing. Eventually, the characterization of a Na-rich SGC is provided.

**Experimental section**

**Synthesis**

The SP-CAM was produced via a spray pyrolysis approach. A precursor solution 0.4 M was prepared by dissolving a stoichiometric amount of NaNO_3_, Mn(NO_3_)_2_·4H_2_O, Fe(NO_3_)_3_·9H_2_O, Co(NO_3_)_2_·6H_2_O, Ni(NO_3_)_2_·6H_2_O and Ti[OCH(CH_3_)_2_]_4_ in distilled water. All reagents employed had a purity grade higher than 98%. The pyrolysis setup consisted of a tubular furnace in which a quartz tube was inserted. One end of the tube was connected to a piezoelectric nebulizer containing the precursor solution, while the other end was connected to a collecting flask. An air stream, made by connecting the collecting flask with a membrane pump, directed the nebulized flux on the flask porous septum. A removable paper filter was placed onto the porous septum to collect the powders coming from the furnace hot region. The furnace was set at 900°C. The resulting powder was placed in a Pt crucible and at 900 °C for 2 hours, followed by rapid cooling.

As for the preparation of the SG-CAM, the following metal precursors were employed: CH_3_COONa (99%), (CH_3_COO)_2_Mn·4H_2_O (99%), (CH_3_COO)_2_Fe (95%), (CH_3_COO)_2_Co·4H_2_O (98%), (CH_3_COO)_2_Ni·4H_2_O (98%) and C_10_H_14_O_5_Ti. First, the titanium precursor was dissolved in 90 mL of water under ultrasonic sonication for 10 min, followed by stirring for 1 h at 50 °C. Then, the other metal precursors were added to the aqueous solution under stirring. Subsequently, citric acid monohydrate (purity: 99%) was added to the resulting solution. After drying at 80 °C overnight, the as-obtained gel was calcinated at 700 °C for 2 h.

**Cathode preparation**

The cathodes were prepared by using 80 wt% of CAM, 10 wt% of carbon black (Ensaco 350P, Imerys), and 10 wt% of a binder (polyvinylidene fluoride, PVdF). The solid content of the slurries was ~26 wt%. Cathodic active materials and carbon powders were mixed in zirconia jars by a planetary ball mill at 150 rpm for 10 min twice, with a rest period of 5 min. The polymeric binder was then added and mixed with a similar procedure. The as prepared mixture was dispersed in N-methylpyrrolidone (NMP) (Sigma-Aldrich) to obtain the slurry, which was cast on a carbon-coated Al foil using a doctor blade with a wet thickness of 300 μm. The electrode was finally dried under vacuum at 40 °C for 14 h to avoid any moisture and oxygen contamination. The cathode (active mass ∼ 2.5 - 3.0 mg cm^-2^) was finally cut into 2 cm^2^ disks and stored in an Ar-filled glove box (MBraun, H_2_O and O_2_ < 0.5 ppm) before electrochemical testing.

All the functional tests were performed using a coin cell type (CR2032 – MTI Corp.) assembled in glove box. Metallic Na was used as the counter electrode. Electrodes were separated with a Whatman^TM^ glass fiber separator, imbibed by 120-150 μL of liquid electrolyte, consisting in a solution 1 M NaPF_6_ in EC:DMC (50:50, by wt.) (E-lyte) added with a 2wt% FEC (Fluoroethylene carbonate).

**Electrochemical tests**

The electrochemical tests were performed by means of potentiodynamic electrochemical impedance spectroscopy (PEIS), galvanostatic cycling with potential limitation (GCPL), and potentiodynamic cycling with galvanostatic acceleration (PCGA) on coin cells assembled as described before. A battery tester Bio-Logic BCS-810 was used. To investigate the evolution of the cell interface upon cycling, PEIS scans between 100 and 0.1 Hz were collected at 50 mV before and at the end of testing.

The electrodes were cycled at room temperature in the voltage range of 1.5 to 4.2 V. Rate performance tests were conducted at a C rate ranging between 0.08C and 1C. Long-term stability tests were conducted at 1C, with two initial cycles at 0.125C, two cycles at 0.25C, followed by two cycles at 0.5C. These initial steps were carried out to allow the SEI formation and stabilization before the long cycling test. A theoretical capacity of 200 mAh g^-1^ was calculated by taking into account the composition and the redox couple actually involved in the electrochemical process.

**Characterizations**

XRD patterns were collected in Bragg-Brentano configuration on a Bruker D6 diffractometer equipped with Cu source and Ni filter. The diffraction patterns were recorded in the 10-70 deg. range with ~0.03 step size. Both powdered and electrode specimens were placed on a dedicated Si zero–background sample holder. The electrodes were sticked on the surface of the sample holder. Possible sample displacement was corrected by taking the Al collector as a reference. XRD data were analysed using the Rietveld method with the software GSAS and its graphical interphase EXPGUI.^[1]^ The Al peaks in the electrodes were modelled with a Le Bail method, as the intensities are strongly affected by texture and no structural information was derived from it. In order to correct for sample displacement, the lattice parameter of Al was set to a reference value (4.049 Å), the lattice parameters of the other phases were refined independently while a sample displacement parameter, common to all the phases investigated, Al included, was varied. The zero-error was fixed during the refinement and set to a standard value.

Raman spectra were recorded by a NTEGRA—Spectra SPM NT-MDT confocal microscope, equipped with MS3504i 350 mm monochromator, and ANDOR Idus CCD, and a solid-state laser operating at 532 nm. Measurements were acquired in reflection mode, using 250 µW power of the laser source at the sample surface to avoid local heating of the sample and annealing effects.

The morphological and compositional characterization of the samples was performed with a Mira3XMU microscope (Tescan) operated at 20 kV and equipped with an EDAX EDX analysis system. The samples were coated with a carbon thin film using a Cressington 208 carbon coater.

ICP-OES analyses were performed by a Avio 220 max (Perkin Elmer), equipped with a AVIO Glass Cyclonic Baffled spray chamber, a quartz torch and dual backside-illuminated charge-coupled device (DBI-CCD) detector. The quantification was carried out on acid-digested solutions in the axial mode at 670.8 nm as wavelength, by an external standard calibration curve. ICP grade multi-elements standard (1000 mg L^-1^, Merck) was diluted to 0.3 – 0.6 – 2.0 – 5.0 – 9.0 mg L^-1^ and then acidified to a final concentration of 2% nitric acid (from ultrapure 65% HNO_3_, Merck). The measurements conditions were as in the following: nebulization gas flow: 0.7 L min^-1^; power RF: 1500 W; auxiliary gas flow: 0.2 L min^-1^; peristaltic pump: 1 mL min^-1^; frequency: 500 Hz. The analysis on the synthesized materials were carried out on previous acid digestion (HNO_3_:HCl solution, 1:3 volume ratio) in a teflon‐lined stainless‐steel reactor and heated at 180°C for 2 h.

**Results and Discussion**

**Configurational entropy**

A quantitative evaluation of S_conf_ requires summing the entropy contributions of all crystallographic sites while accounting for their respective multiplicities. Assuming the anion site is fully occupied and does not contribute, the configurational entropy of both O3 and P3 polymorphs can be calculated using the same expression, since both cation sites have identical multiplicity:

$$S_{conf}=-R\left( \sum_{i,Na} \chi_{i}\ln\chi_{i}+\sum_{i,TM} \chi_{i}\ln\chi_{i} \right)$$

where $\chi_{i}$ denotes the molar fraction of each species sharing a given crystallographic site, in this case those related to Na and TM ions. The Na site actively contributes to the magnitude of S_conf_ when the structure is not fully sodiated, as configurational entropy arises from the distribution of Na^+^ ions and vacancies. This contribution reaches its maximum for x=0.5 in Na_x_TMO_2_. According to the overall compositions of the specimens (Na_0.52_Ti_0.19_Mn_0.19_Fe_0.21_Ni_0.21_Co_0.20_O_2_ for spray pyrolysis and Na_0.57_Ti_0.19_Mn_0.20_Fe_0.20_Ni_0.21_Co_0.20_O_2_ for sol-gel), the corresponding S_conf_ values are 2.30 R and 2.29 R, respectively.

These values, however, do not consider phase partitioning. As discussed in the Supporting Information, the actual composition of the active layered phase in the spray-pyrolyzed sample is approximately Na_0.80_Ti_0.23_Mn_0.23_Fe_0.11_Ni_0.17_Co_0.13_O_2_, giving S_conf_ = 2.03 R. Using the same procedure the active phase composition in the sol–gel sample is estimated as Na_0.63_Ti_0.22_Mn_0.22_Fe_0.12_Ni_0.23_Co_0.21_O_2_, which leads to S_conf_ equal to 2.24 R. Both values exceed 1.5 R, a commonly cited heuristic threshold for high-entropy oxides.

We stress, however, that these estimates may still not reflect the true configurational entropy. Determining the actual S_conf_ would require precise knowledge of the chemical composition of each polymorph, in particular the exact distribution of cations between the two layered phases. Such information cannot be directly obtained using conventional characterization methods. Consequently, the real S_conf_ could be either higher or lower than our estimates.

Finally, we note that the Na-site contribution to S_conf_ is intrinsic to binary Na_x_TMO_2_ compounds and evolves continuously during battery operation as x changes. Therefore, when comparing configurational entropy among different cathode active materials, the contribution from the TM site alone provides a more meaningful basis for comparison.

**Composition**

The composition was determined on the powdered specimens. The fraction of transition metals was determined through EDS analysis. Owing to the limited sensibility of this technique towards light elements, the fraction of Na was determined through ICP. Table S1 summarizes the chemical composition of the CAMs.

**Table S1**. Composition of the cathode under investigation derived from ICP-OES analysis (for Na content) and Energy Dispersive X-ray Spectroscopy (EDX), concerning the transition metals.

| **Element** | **SP-CAM** | **SG-CAM** |
| --- | --- | --- |
| Na | 0.52 | 0.57 |
| Ti | 0.19 | 0.19 |
| Mn | 0.19 | 0.20 |
| Fe | 0.21 | 0.20 |
| Ni | 0.21 | 0.21 |
| Co | 0.20 | 0.20 |
| **Composition** | Na_0.52_Ti_0.19_Mn_0.19_Fe_0.21_Ni_0.21_Co_0.20_O_2_ | Na_0.57_Ti_0.19_Mn_0.20_Fe_0.20_Ni_0.21_Co_0.20_O_2_ |

**μ Raman Spectroscopy**

**Cathode active materials**

The spatial homogeneity of the CAMs was evaluated by micro-Raman spectroscopy (μRS).


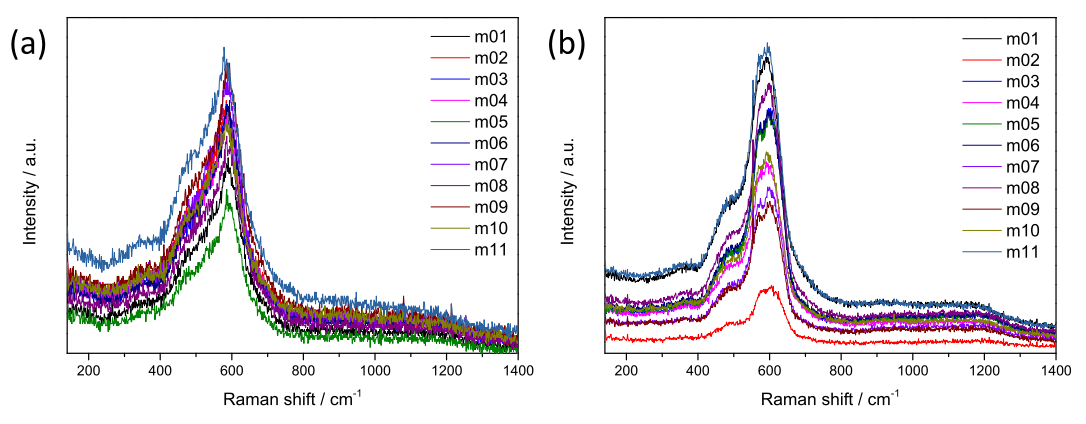


**Figure S1**. Micro-Raman spectra measured at randomly selected positions in (a) SP- and (b) SG-CAMs

The lack of shifts and changes in relative intensities of the bands in the spectra recorded at different random positions in each specimen revealed that both samples are spatially uniform. (Fig. S1)

**Electrodes**

Figure 3gh in the main text displays portions of the average spectra of the electrodes. Figures S2 and S3 show the spectra recorded at different randomly selected positions in each electrode, both at open circuit voltage (OCV) and after stability tests. The comparison proves that both electrodes are spatially uniform and that the cycling does not alter their homogeneity.


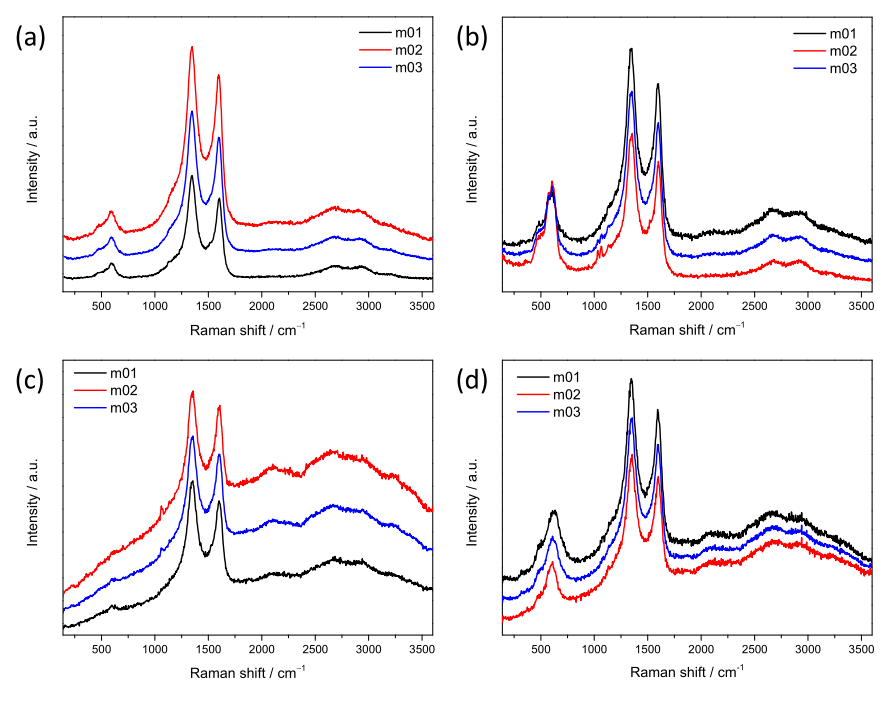


**Figure S2.** Micro-Raman spectra measured at randomly selected positions in (a,c) SPC and (b,d) SGC. Data refer to the electrodes (a,b) at open circuit voltage and (c,d) at the end of stability tests. The use of a 600 lines mm^−1^ grating allows probing the spectral region were the phonon modes arising from the carbonaceous additive are detected.

In the spectra obtained as average of those recorded using a 600 lines mm^-1^ grating (Figure S2), the Raman fingerprint of the amorphous carbonaceous additive is visible in the higher frequency region of the spectra (> 1000 cm^–1^), namely the D- and G-bands at ~1346 and ~1590 cm^–1^, respectively, and overtones and combination bands at higher frequencies.^[2,3]^ At the end of cycling, these bands appear superimposed on a broad and relatively intense photoluminescence background, which hints at the increase of the defect density in the carbonaceous matrix.^[4]^ Moreover, in the SPC the relative intensity of the layered oxide modes (< 1000 cm^–1^) decreases after the stability test, suggesting the possible detachment of part of the active material from the electrode, possibly during the extraction of the electrode from the battery. A similar issue has been encountered in the XRD investigation.


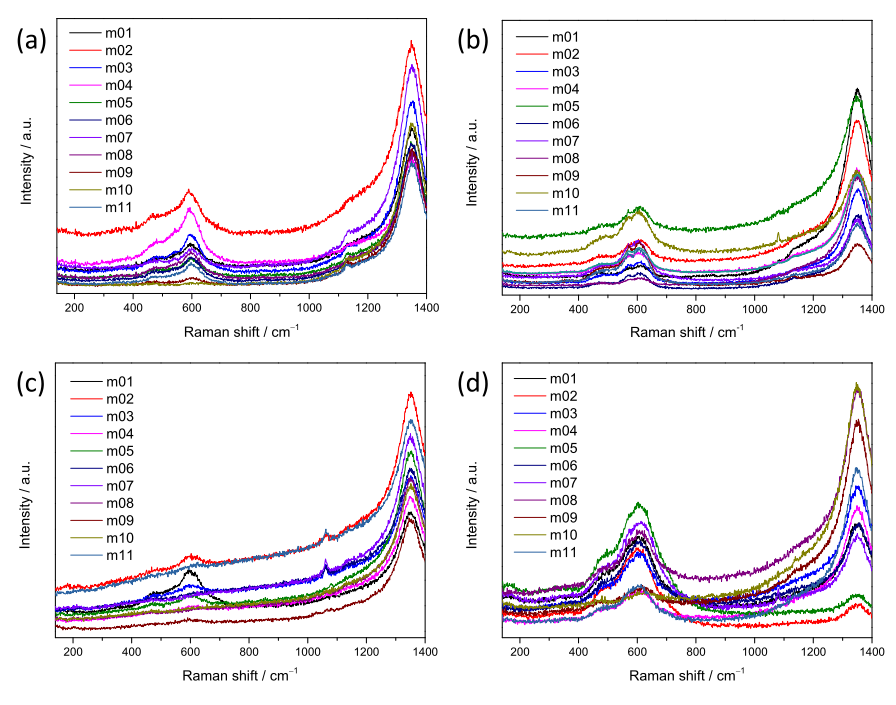


**Figure S3.** Micro-Raman spectra measured at randomly selected positions in (a,c) SPC and (b,d) SGC. Data refer to the electrodes (a,b) at open circuit voltage (OCV) and (c,d) at the end of stability tests.


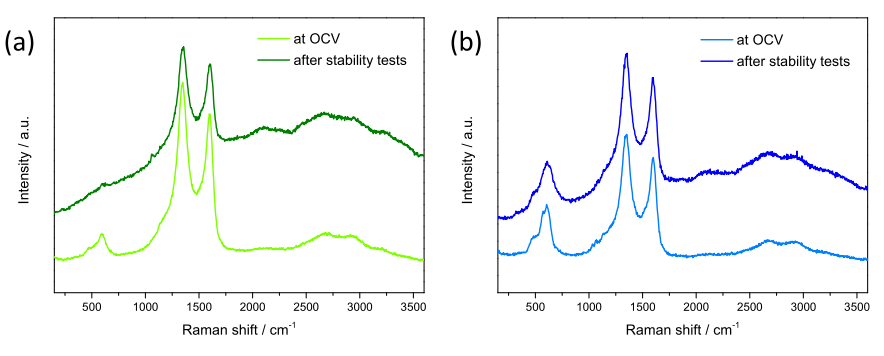


**Figure S4.** Comparison between averaged micro-Raman spectra of electrodes at OCV and at the end of stability tests for (a) SPC and (b) SGC.

Figure 3e-f in the main text shows the region of the layered-oxide modes of the averaged spectra of both cathodes before (OCV) and after stability tests. The spectra of both fresh cathodes do not substantially differ from those of the pristine active material (not shown for briefness). Similarly, the spectra measured at the end of the stability test substantially follow the Raman fingerprint of the pristine oxides, which is an indication of the structural stability of the material. In the case of the SPC, after prolonged cycling, only very slight intensity changes are observed in the region of the *E*_g_ O‒M‒O bending mode, while the *A*_1g_ M‒O stretching mode of the P3/O3-structure becomes less resolved. These findings hint at the occurrence of small changes in the relative amounts of the two phases. A more marked intensity increase is observed on the higher-frequency tail of the M‒O stretching mode of the P3/O3-structure. Similar changes have been reported also by other authors and suggest an increase in spinel-structured impurities.^[5]^ Conversely, no appreciable difference is observed between the spectra of the electrodes extracted from the batteries at OCV and after the initial low-rate cycles (Figure S5b), which indicates that the P3/O3 to spinel phase transformation occurs during the subsequent cycles at 1C rate. These changes are not appreciable in the SPC, confirming the stability of the cathode.


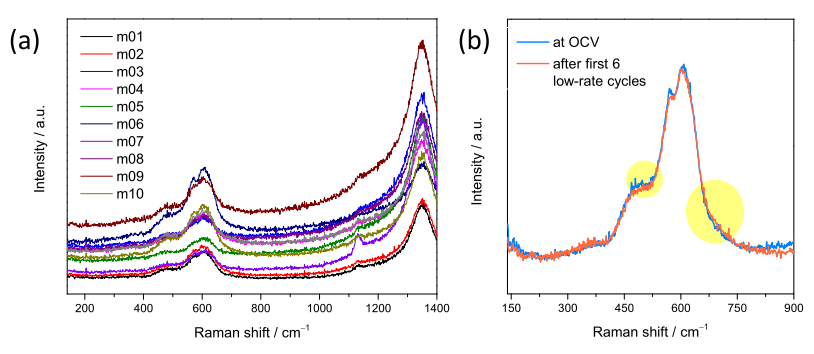


**Figure S5.** (a) μRS spectra measured at randomly selected positions in SG extracted from the battery after the initial low-rate cycles (two cycles at 0.125C + two cycles at 0.25C + two cycles at 0.5C). (b) Resulting averaged spectrum compared with that measured at OCV.

**X-Ray Diffraction**

| 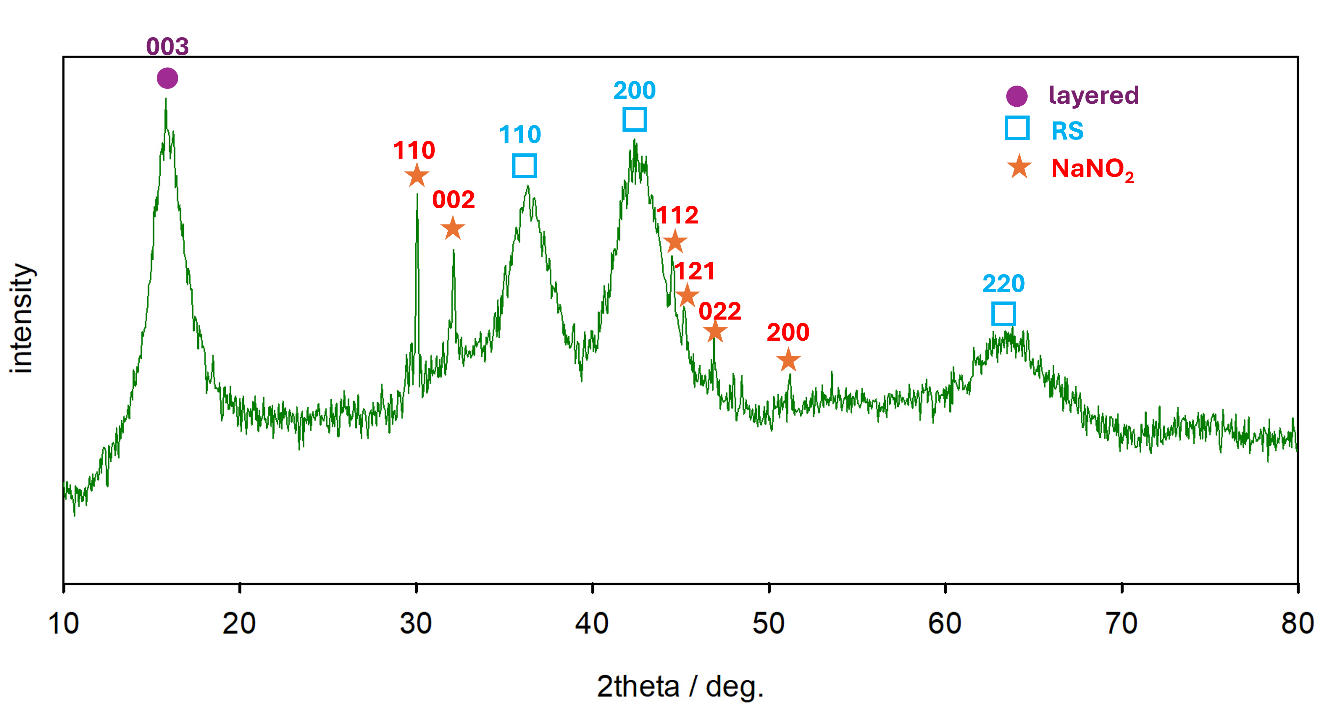 |
| --- |
| **Figure S6**. Experimental XRD pattern of the precursor of the SP-CAM after the pyrolysis without further annealing. |

Figure S6 reports the XRD pattern collected on the CAM obtained by spray pyrolysis without further annealing at 900°C. Blurred peaks are accompanied by some sharp reflections. The first peak corresponds to the main reflection (003) of layered phases, either O3 or P3. The other three broad peaks are consistent with the first reflection of a rock-salt phase. As they are intense and very broad, they likely overlap with the less intense reflections of the layered compound(s). Such an overlap of poorly defined features prevents an accurate modelling of the experimental pattern. The sharp peaks, evidence of a minority phase, are consistent with the orthorhombic structure of NaNO_2_, space group *Imm*2, lattice parameter *a*~3.56 Å, *b*~5.38 Å, *c*~5.57 Å. NaNO_2_ reflections were observed systematically in the synthesis with different levels of Na and different stoichiometry. NaNO_2_ likely derives from the NaNO_3_ precursor, which at high temperature decomposes to produce NaNO_2_ and molecular oxygen. Sodium nitrite should then decompose to form Na_2_O, but the pyrolysis process takes only few seconds and reactions do not occur completely.^[6]^ The occurrence of the secondary phase in the precursor of the SP-CAM was often accompanied by the presence of regular crystals observed from SEM, see *e.g.* in Fig. S7:

| 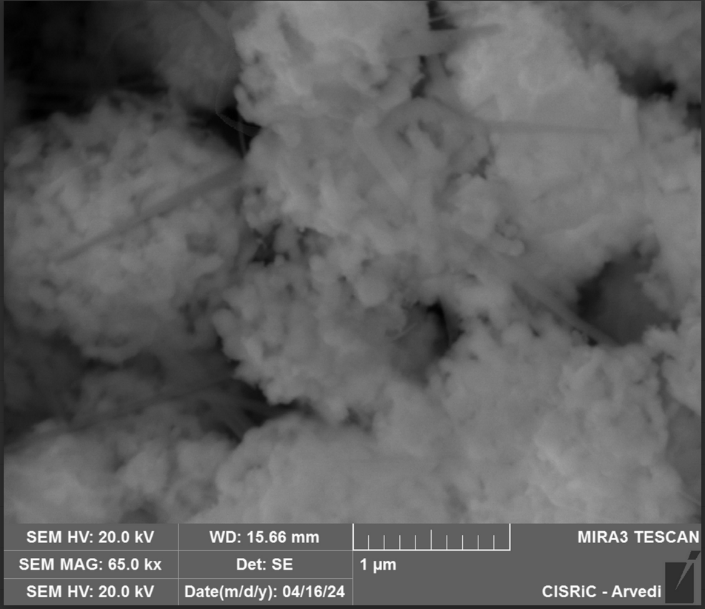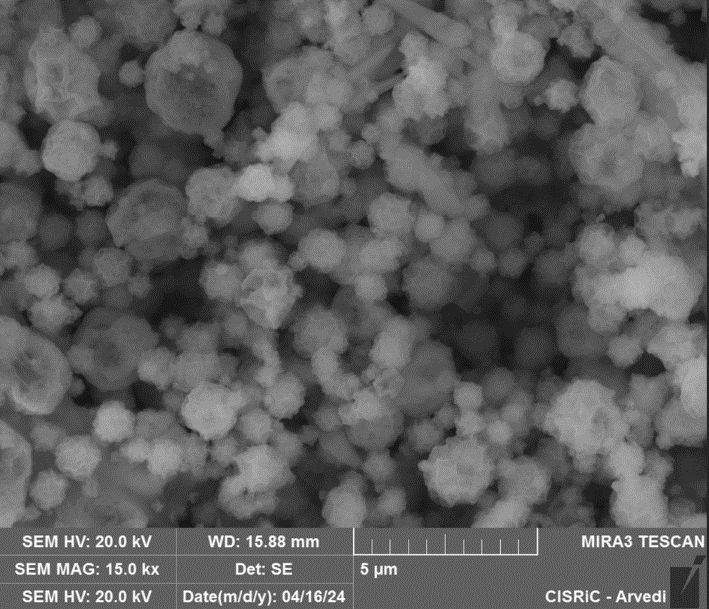 |
| --- |
| **Figure S7**. SEM images of precursor of SP-CAM produced by spray pyrolysis between 700 and 900 °C showing the presence of secondary phases corresponding to NaNO_2_. |

**Cathode active materials**

Figure S8 reports Rietveld refinement plots on the SP- and SG-CAMs. The corresponding phase fractions computed via Rietveld refinements are reported in Table S2.

| 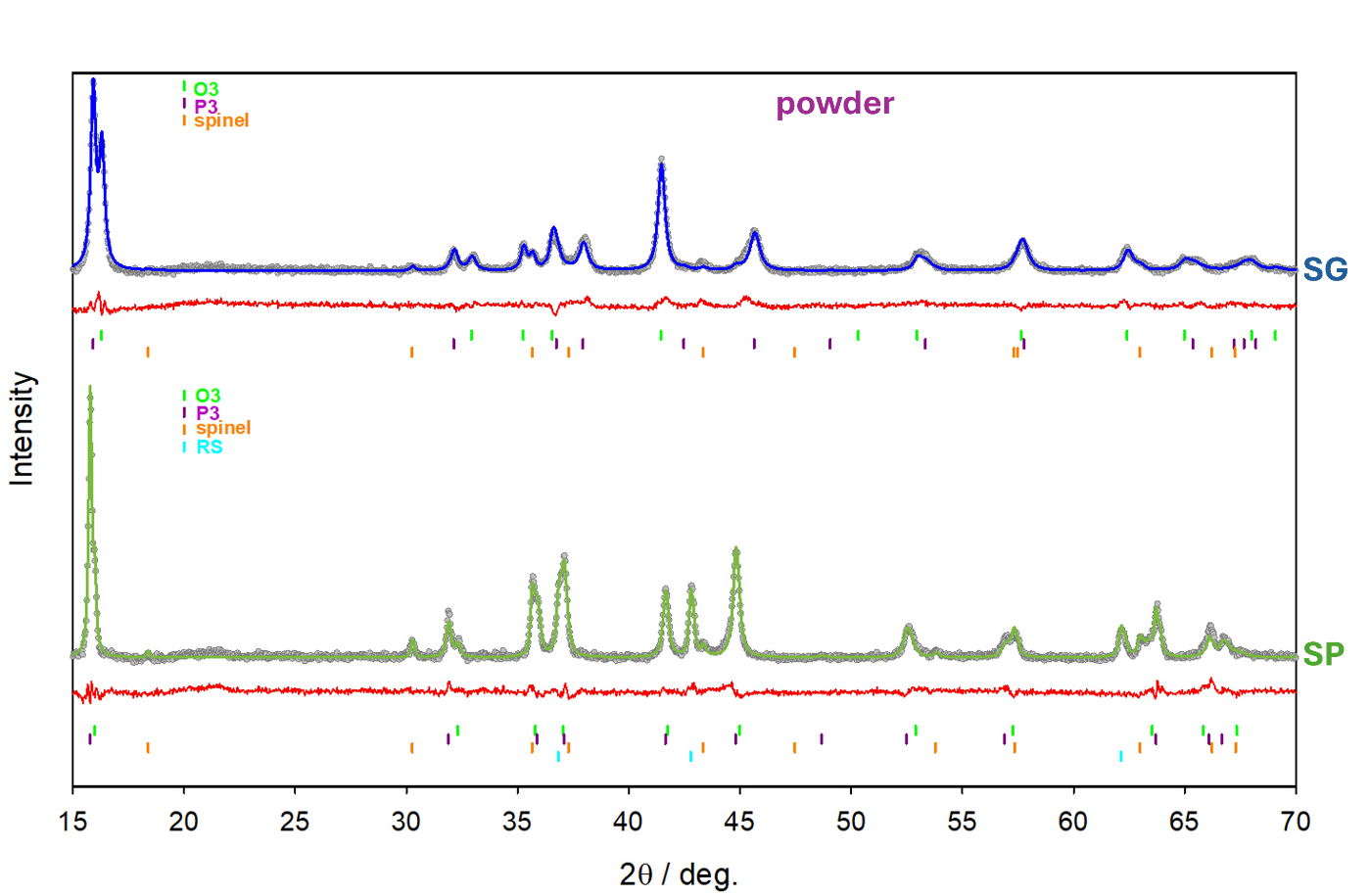 |
| --- |
| **Figure S8**. Rietveld refinements on the powdered SP- (green) and SG-CAM (blue). Gray dots: experimental, solid lines: calculated profile, red solid lines: difference curve. The position of the reflections of all phases considered in the fit are reported with bar below the plots, according to the color scale in the graph. |

**Table S2**. Phase fraction and cell parameters of SP- and SG-CAMs computed from Rietveld refinements.

| SP-CAM | %wt. | *a* / Å | *c* / Å |
| --- | --- | --- | --- |
| O3 | 19.3 | 2.9326(4) | 16.583(3) |
| P3 | 59.5 | 2.9179(2) | 16.806(3) |
| spinel | 7.9 | 8.3389(4) | - |
| rock-salt | 13.3 | 4.2194(3) | - |

| SG-CAM | %wt. | *a* / Å | *c* / Å |
| --- | --- | --- | --- |
| O3 | 55.0 | 2.9739(3) | 16.306(3) |
| P3 | 39.8 | 2.8525(3) | 16.703(4) |
| spinel | 5.2 | 8.394(3) | - |

**Composition of the layered active phases of the SP-CAM**

The overall composition of the CAM as probed by combining ICP for Na and EDS for the transition metals TMs is Na_0.52_Ti_0.19_Mn_0.19_Fe_0.21_Ni_0.21_Co_0.20_O_2_. Na is known not to intercalate into spinel nor RS phases, as Na^+^ ions cannot be accommodated into those structures. The Na^+^ ionic radius, compared to those of TMs, is simply too big. It follows that Na accumulates preferentially into the layered phases P3 and O3 and its global content will be, as a consequence, larger than 0.52 per formula unit. The composition of the layered phases will differ also in respect to the TMs. Indeed, the secondary phases will be likely rich in one or some of the elements constituting the CAM. The RS phase is composed of divalent cations only. Trivalent cations would fit it balanced by Na^+^ ions, which are, however, too big to form a rock salt phase. It follows that the RS, at ambient conditions, is composed of Ni and Co, which are the two cations stable in the 2^+^ state. Kubbon and Hu reported the evolution of the lattice parameter in the whole NiO-CoO solid solution, providing an equation to relate the observed RS lattice parameter to the content of NiO:^[7]^

$$a=-0.0282NiO+4.2581$$

The observed cell parameter (4.2194 Å) is related to a fraction of NiO equal to 0.47, thus leading to composition Ni_0.47_Co_0.53_O for the RS phase. As concerns the spinel phase, in principle, it can host combination of all TM ions. Limiting the search to the single TM oxides, at ambient conditions, only Fe_3_O_4_ and Co_3_O_4_ exhibit the cubic spinel structure, while Mn_3_O_4_ crystallizes with a distorted spinel structure, which is not observed here. The observed lattice parameter (~8.339 Å) is close to the value of magnetite Fe_3_O_4_ (~8.39 Å), while Co_3_O_4_ has a much smaller unit cell (~8.08 Å). Hence, the secondary phase could be composed of Co-doped Fe_3_O_4_. However, we cannot exclude that the secondary phase observed is maghemite (Fe_2_O_3_) rather than magnetite, which is characterized by the same structural motive and a smaller unit cell (~8.336 Å). For simplicity, in the manuscript we refer to “spinel” since maghemite has a lower symmetry than spinel (space group *P*4_3_32), but superstructure peaks have low intensity that are arduous to be resolved from the background, even in pure specimens. We will assume that this phase is composed only of Fe ions among TMs.

Table S3 reports the weighted phase fractions computed from Rietveld refinement against the pattern of the SP-CAM. From these fractions it is possible to estimate the overall composition of the layered phases excluding the ions that segregate into secondary phases. To this purpose, we have to explicit the number of ions per formula unit which are segregated into the secondary phases, therefore, molar phase fractions have to be considered. The unit cell formula weights of the layered phases reported in Table S3 are estimated based on the TMs content estimated by SEM-EDS (Table S1). In addition, we assumed that spinel is made only of Fe (as TM) and only Co and Ni contribute, as detailed above, to the RS phase. The last row in Table S3 provides the number of moles of each phase in 100 g of the SP-CAM.

**Table S3**. Conversion from mass fraction to mol content for all phases observed in the SP-CAM.

| phase | O3 | P3 | spinel | RS |
| --- | --- | --- | --- | --- |
| %wt. | 19.3 | 59.5 | 7.9 | 13.3 |
| unit cell weight (g/mol) | 330.76 | 326.87 | 1852.3 | 326.87 |
| mol | 0.0583 | 0.1820 | 0.004265 | 0.04069 |

Table S3 lists the number of moles of each TM element within each of the phases for 100 g of CAM. As we cannot resolve the elements within each of O3 and P3 layered phase, the two polymorphs are considered here as a single entity. The global number of moles is obtained by multiplying the sum of the moles of the layered phases by Z (=3) and by the overall fraction of each TM as derived from SEM-EDX. The number of moles of each element contributing to the layered compounds is obtained by subtracting the moles accumulated into the single secondary phase by the global content. The column “norm layered” of Table S4 reports the fraction of TMs normalized to unity into the layered phases. This normalized fraction corresponds to the one expressed in the formula unit Na_x_TMO_2_.

**Table S4**. Number of moles of each TM estimated for spinel, rock salt and layered polymorphs.

| element | global | Sp | RS | layered | norm layered | norm layered adjusted |
| --- | --- | --- | --- | --- | --- | --- |
| Ni | 0.151 | - | 0.076 | 0.075 | 0.165 | 0.169 |
| Fe | 0.151 | 0.102 | - | 0.049 | 0.108 | 0.112 |
| Mn | 0.137 | - | - | 0.137 | 0.300 | 0.293 |
| Co | 0.144 | - | 0.086 | 0.057 | 0.126 | 0.132 |
| Ti | 0.137 | - | - | 0.137 | 0.300 | 0.293 |

It should be noted that, if this is the correct composition of the layered phases, the above calculation should be iterated as the unit cell phase fractions would slightly change, as listed in the last column. The changes, however, do not exceed 0.6 percent unit for any TM. Finally, we note that repeating the calculation with maghemite instead of magnetite leads to the same results.

Concerning the Na amount, the compositional analysis states that its content is 2.7 times larger than those of Mn and Ti. Supposing that those TM are distributed only within the layered phases, the overall content of Na within the layered phases amount to ~0.80 per formula unit. This is, however, distributed between the P3 (in excess) and the O3 phases. The determination of the distribution of Na among the two layered polymorphs is an ill-defined problem. However, assuming that the O3 phase is fully sodiated, and knowing that the total fraction of Na is 0.8 per formula unit, the fraction of Na in the P3 polymorph is 0.735, as given by resolving:

$$0.245\cdot1+0.755\cdot{xNa}_{P3}=0.8$$

Similary, the overall composition of the active layered phases can be estimated for the SG-CAM as well. In this case, the only secondary phase is spinel, therefore the layered phases experience a depletion only in the content of Fe, as listed in Table S5. The average Na content among the two phases amounts to 0.62.

**Table S5**. Number of moles of each TM estimated for spinel and layered polymorphs.

| element | global | Sp | layered | norm layered | norm layered adjusted |
| --- | --- | --- | --- | --- | --- |
| Ni | 0.151 | - | 0.075 | 0.151 | 0.232 |
| Fe | 0.144 | 0.067 | 0.049 | 0.077 | 0.118 |
| Mn | 0.144 | - | 0.137 | 0.144 | 0.221 |
| Co | 0.144 | - | 0.057 | 0.144 | 0.221 |
| Ti | 0.137 | - | 0.137 | 0.137 | 0.210 |

**Electrodes**

Figures S9-10 and Table S6 report plots and crystallographic parameters of the electrodes. Note that in all the plots a sharp feature at ~26 deg. has been removed. This feature, evident in all XRD patterns collected on cathodes, even fresh ones, was already observed by our group in electrodes with different compositions, even Li-based, when the cathode is produced using some components as in this study. Note also that no peak of RS, spinel and layered phase is expected in the removed angular range. It is clear from the Rietveld plots that spent electrodes show more blurred features. This is partially due to the mechanical degradation of the cathode, which reduces the amount of active material suitable for the investigation and makes the cathode more corrugated. This reflects on the signal of all peaks, including those of the Al reference which did not undergo any change. This is evident in the SPC, where the main Al peak shows some broadening. According to Table S6, the change in composition of the SPC during electrochemical testing affects only the layered phases (full transformation of P3 into O3) and is coherent with a larger amount of Na, compared to the original state, observed at the end of the stability electrochemical process.

| 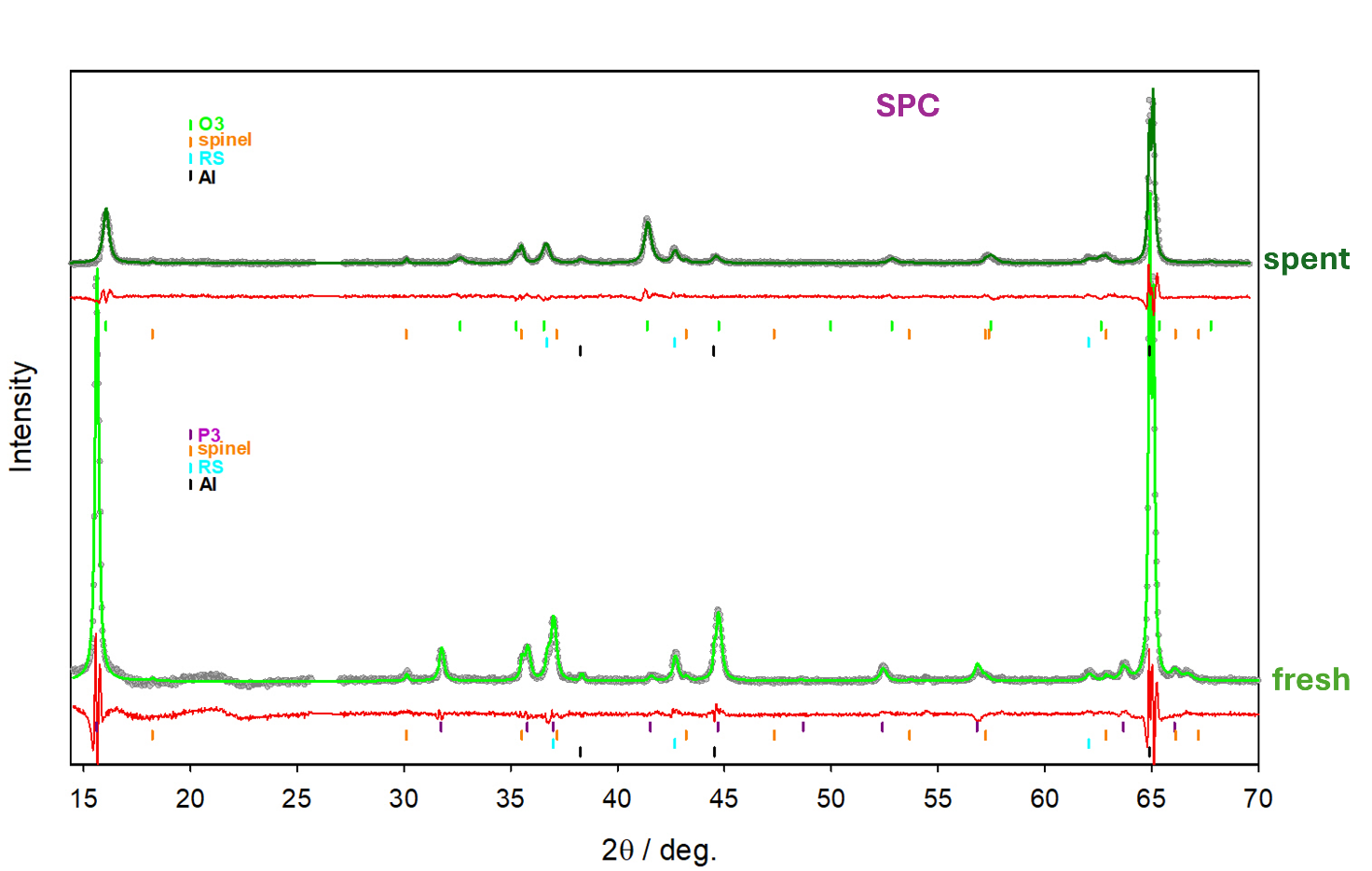 |
| --- |
| **Figure S9**. Rietveld refinements on the SPC in fresh (light green) and spent (dark green) conditions. Gray dots: experimental, solid lines: calculated profile, red solid lines: difference curve. The position of the reflections of all phases considered in the fit are reported with bar below the plots, according to the color scale in the graph. |

Concerning the SGC, the peaks broaden more than for the SPC, and the broadening affects less the signal of Al collector, thus suggesting that the peak broadening is related to physical changes occurring in the CAM rather than in the shape of the cathode itself. In addition, the peak broadening is more evident for the O3 phase, which, according to Table S6, shows an impressive contraction of lattice parameter c (15.56 Å compared to 16.31 Å in the powder specimen). This is consistent either with a very large fraction of Na, suggesting that when the layered compound is fully sodiated, that part of Na remains trapped in the O3 phase, and/or with a change of composition of the active layered phases. In this case, the O3 would undergo an enrichment in smaller ions. Concerning the spinel phase, it is clearly present, as few peaks are visible. However, due to the strong overlap with the broad reflections of the layered phases, especially O3, the phase quantification is not reliable, also as suggested by the large standard uncertainty computed by the fit. Whatever the absolute value of the spinel content, we can state the phase is present and likely increased in fraction after the electrochemical cycling, as evidenced by the μRS.

| 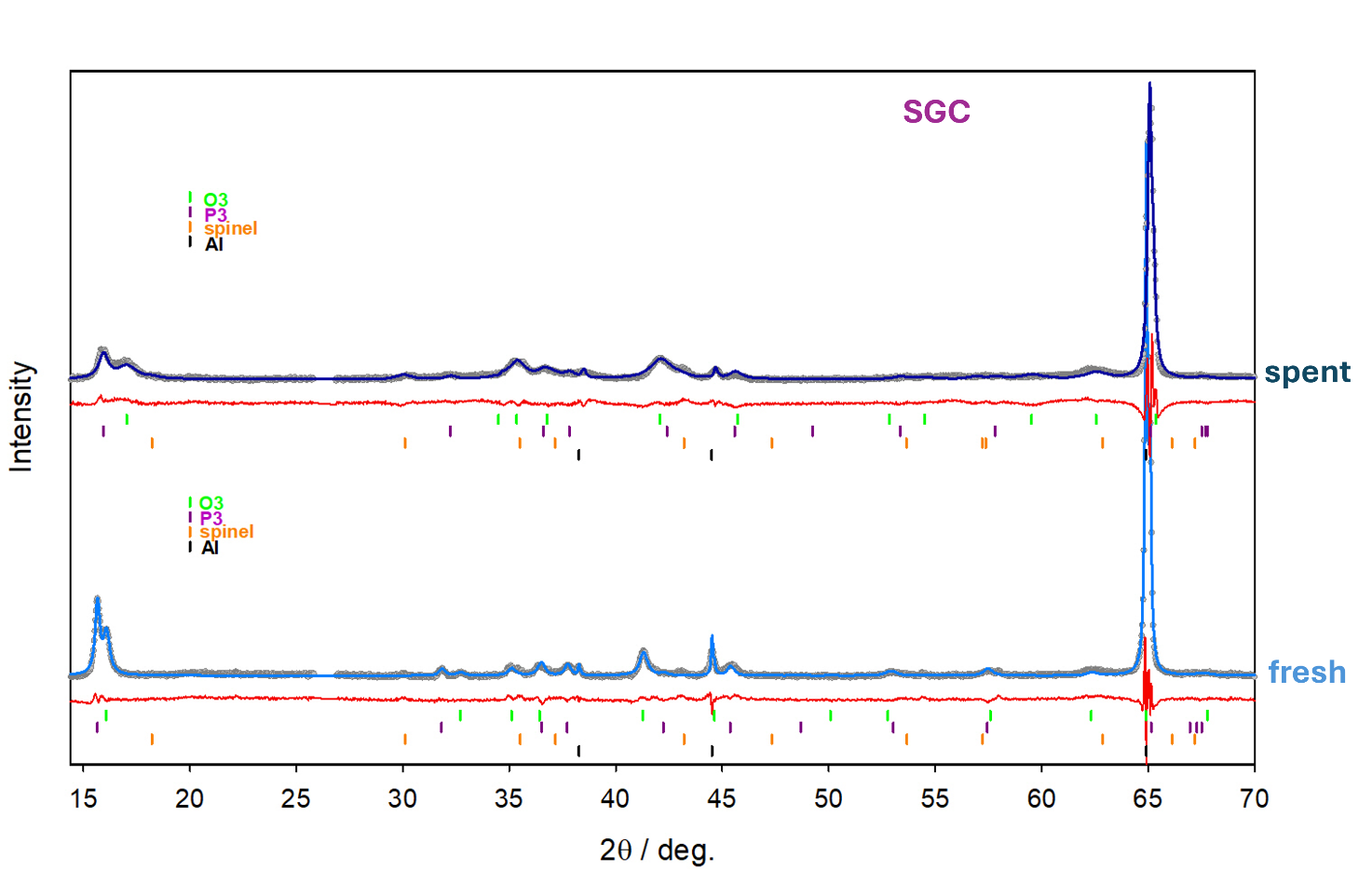 |
| --- |
| **Figure S10**. Rietveld refinements on the SGC in fresh (light blue) and spent (dark blue) conditions. Gray dots: experimental, solid lines: calculated profile, red solid lines: difference curve. The position of the reflections of all phases considered in the fit are reported with bar below the plots, according to the color scale in the graph. |

| 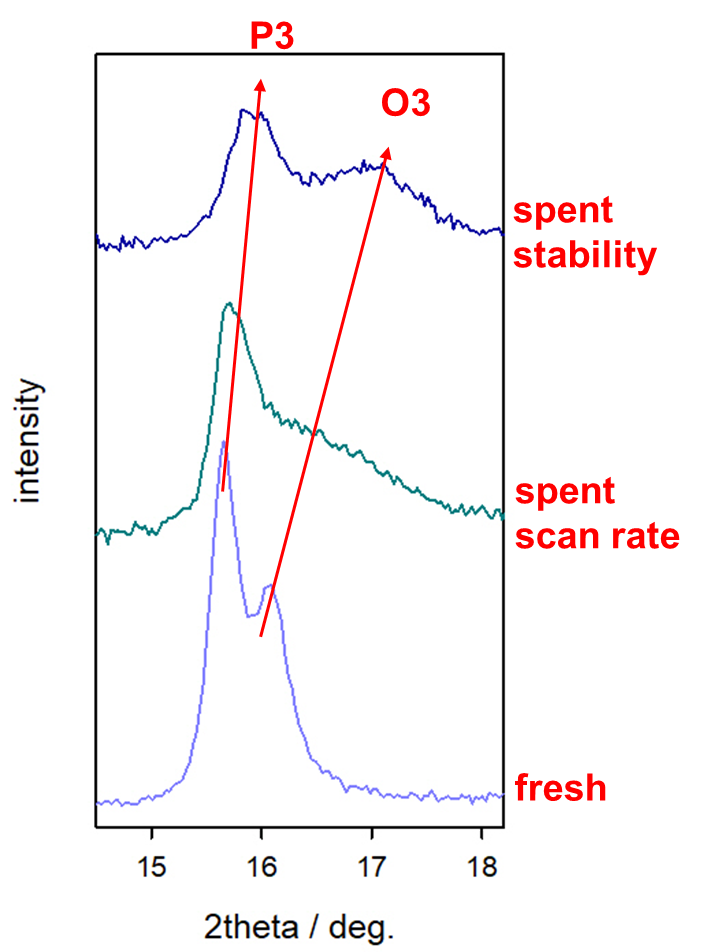 |
| --- |
| **Figure S11**. Evolution of the main 003 reflections of the P3 (left) and O3 (right) layered polymorphs from the experimental XRD patterns of the SGC in fresh condition (OCV), after the long-term stability test (300 cycles at 1C rate) and after the scan rate (Figure 2b).  The two polymorphs exhibit a different structural evolution after the two different electrochemical cycling, especially with respect to the O3 phase. For both types of electrochemical cycling, the peaks undergo important peak broadening, especially for the O3 phase. Such a broadening is more pronounced after the scan rate process, testifying an important loss of structural coherence. |

**Table S6**. Phase fraction and cell parameters of spent SPC and SGC computed from Rietveld refinements.

| SPC | %wt. | *a* / Å | *c* / Å |
| --- | --- | --- | --- |
| O3 | 79.9(5) | 2.9552(4) | 16.347(3) |
| spinel | 8.7(8) | 8.331(2) | - |
| rock-salt | 11.8(6) | 4.2152(7) | - |

| SGC | %wt. | *a* / Å | *c* / Å |
| --- | --- | --- | --- |
| O3 | 54(1) | 2.966(2) | 15.56(2) |
| P3 | 17(1) | 2.861(3) | 16.63(1) |
| spinel | 29(5) | 8.40(1) | - |

**Electrochemistry**

Here full electrochemical data of SPC and SGC are reported. Figure S12 displays the first four cycles of the PCGA curves, while Figures S13-S14 compare the specific capacities during charge and discharge. The performance of the electrodes reported in this study are compared to those reported in the literature for Na-based high entropy oxides in Table S6.


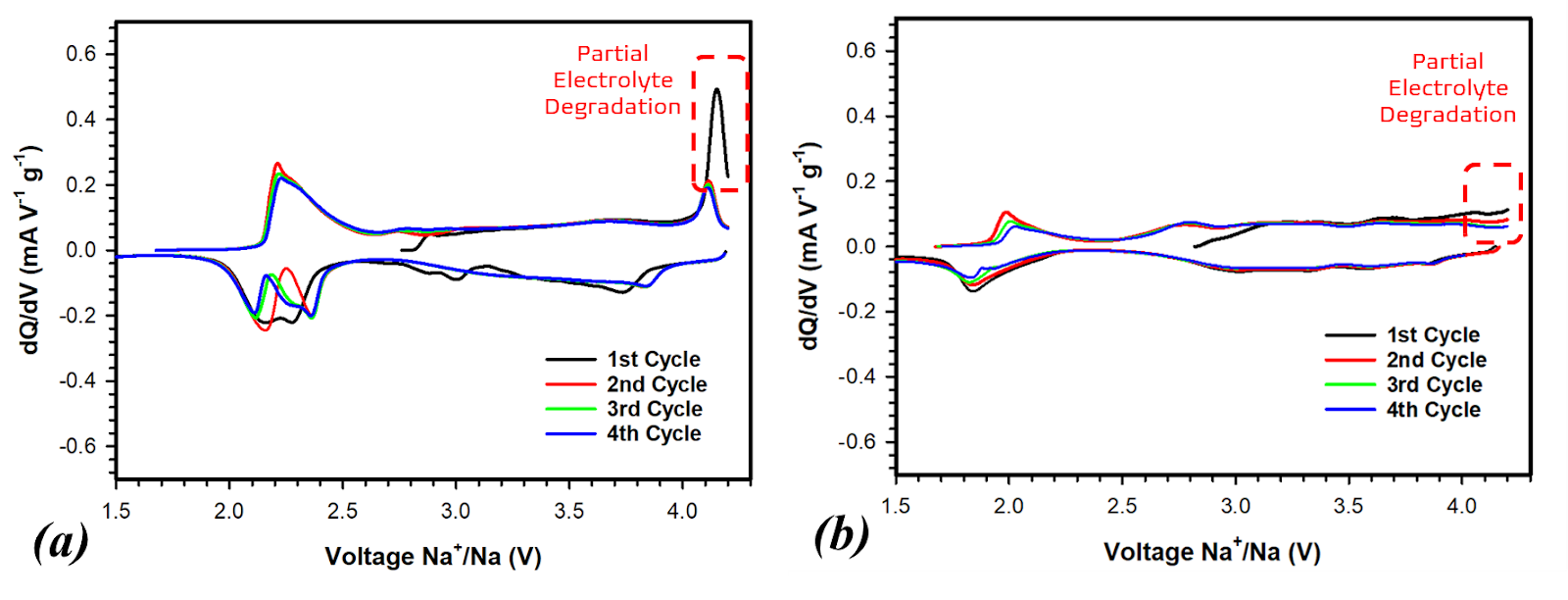


**Figure S12*.***  dQ/dV curves for the first four cycles performed on (a) SPC and (b) SGC between 1.5 V and 4.2 V at 0.08 C.


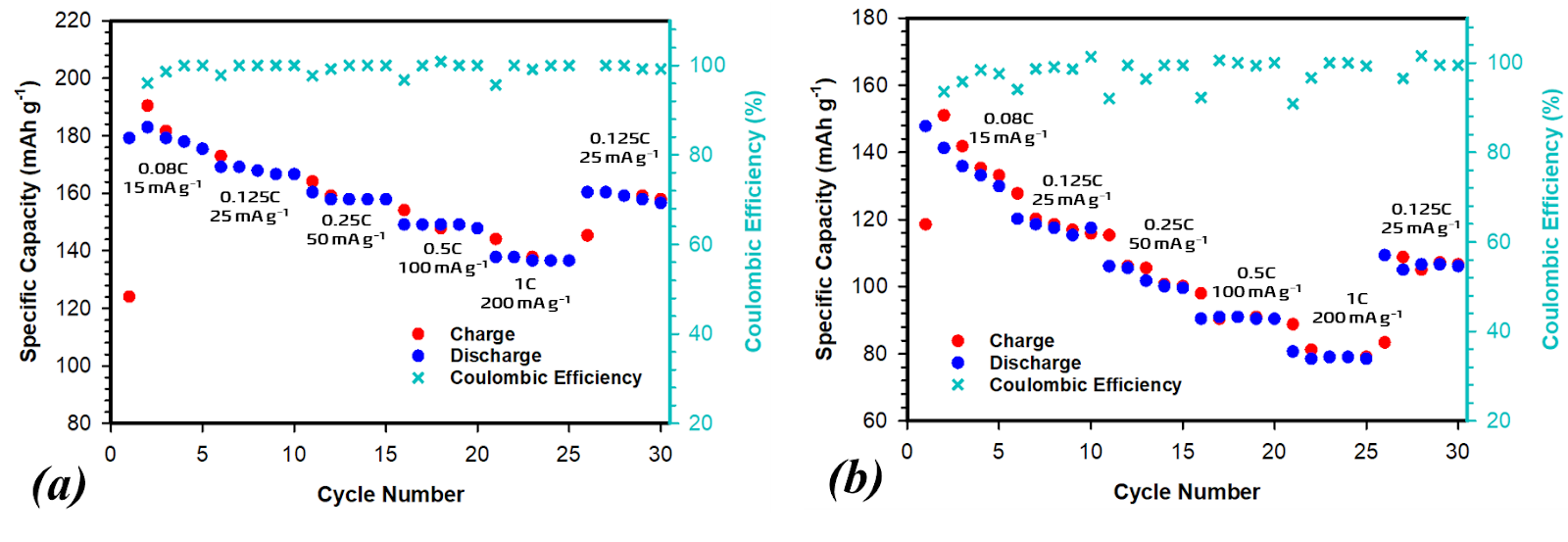


**Figure S13.** Comparison between charge and discharge capacity, coulombic efficiency CE and rate capability test at different current densities for (a) SPC and (b) SGC


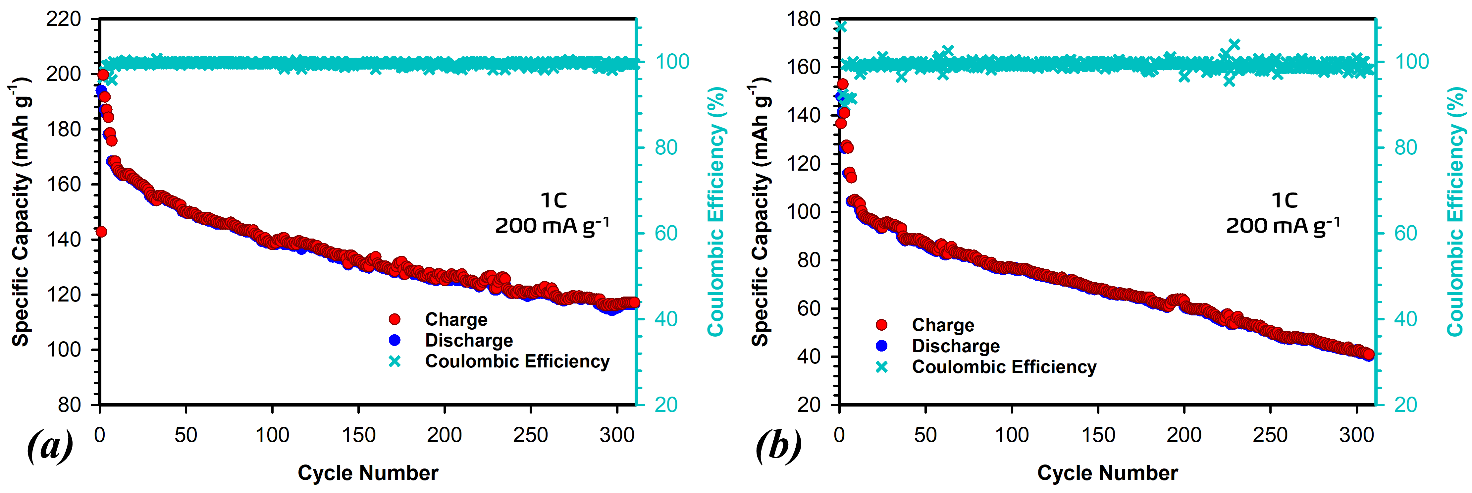


**Figure S14.** Long-term cycling performed at 1C (200 mA g^-1^) comparing charge and discharge for a) SPC and b) SGC, in a working voltage range from 4.2 to 1.5 V.

**Table S7**. Comparison of the performance of the cathodes of this study with some literature reports.

| **Cathode** | **Rate performance** | | **Cycling performance** | | | | | **Ref.** |
| --- | --- | --- | --- | --- | --- | --- | --- | --- |
|  | **Discharge capacity (mAh g^−1^) @ low C rate** | **Discharge capacity (mAh g^−1^) @ high C rate** | **C Rate** | **Initial Discharge capacity (mAh g^−1^)** | **cycles #** | **Final Discharge capacity (mAh g^−1^)** | **Retention (%)** |  |
| **SPC** | ~180 @ 0.08C | ~138 @ 1C | 1C | ~168 | 300 | ~118 | ~69 | This work |
| **SGC** | ~135 @ 0.08C | ~79 @ 1C | 1C | ~105 | 300 | ~40 | ~38 | This work |
| NaMn_0.2_Fe_0.2_Co_0.2_Ni_0.2_Ti_0.2_O_2_ | 193 @ 0.1C | 118 @ 1C | 0.1C |  | 100 | 167.56 | 97 | [8] |
| NaMn_0.2_Fe_0.2_Co_0.2_Ni_0.2_Sn_0.1_Al_0.05_Mg_0.05_O_2_ | 152 @ 0.1C | 112 @ 1C | 0.5C | 142 | 300 | 85 | 60 | [9] |
| NaMn_0.2_Fe_0.2_Co_0.2_Ni_0.2_Ti_0.2_O_2_ | 193 @ 0.1C | 126 @ 1C |  |  |  |  |  | [9] |
| NaMn_1/6_Fe_1/6_Co_1/6_Ni_1/6_Ti_1/6_Cu_1/6_O_2_ | 143 @ 0.1C | 65 @ 1C |  |  |  |  |  | [9] |
| Na(Ni_0.3_Fe_0.2_Mn_0.5_)_0.85_Ti_0.1_Co_0.05_O_2_ | 174.7 @ 0.1C | 127.6 @ 1C | 1C |  | 300 | 106.7 | 78 | [10] |
| Na_0.8_[Ni_1/5_Fe_1/5_Co_1/5_Mn_1/5_Ti_1/5_]O_2_ | 107 @ 0.05C | 94.1 @ 5C | 0.05C | ~107 | 100 | 96.62 | 90 | [11] |
| Na_7/9_Ni_2/9_Mn_4/9_Fe_1/9_Cu_1/9_Ti_1/9_O_2_ | 157.85 @ 0.1C | 84.41 @ 10C | 1C |  | 200 | 88.92 | 75.35 | [12] |
| Na(MgCu)_1/12_(NiCoFeMnTi)_1/6_O_2_ | 146.6 @  10 mA g^−1^ | 128.3 @  100 mA g^−1^ | 100 mA g^−1^ |  | 700 | 108 | 86.5 | [13] |
| NaNi_0.3_Cu_0.1_Fe_0.2_Mn_0.2_Ti_0.2_O_2_ | 122.8 @ 0.1C | 99.5 @ 1C | 1C |  | 200 | 83.1 | 77.16 | [14] |
| NaMg_0.08_Cu_0.12_Ni_0.2_Fe_0.2_Mn_0.2_Ti_0.2_O_2_ | 131.1 @ 0.1C | 123.8 @ 1C | 1C |  | 200 |  | 84 | [15] |

**X-Ray Absorption spectroscopy**

Figure S15 shows the normalized XANES fluorescence spectra collected at the Co, Fe and Mn K edge of the fresh SPC. The edges were selected the three elements are generally stable under different oxidation states. Comparisons with reference standards suggest that Fe is mainly in 3^+^ state, Mn in 4^+^ state, while Co has a mixed 2^+^/3^+^ state. Ni and Ti are assumed to be in their conventional 2^+^ and 4^+^ state, respectively. Figure S16 compared XANES fluorescence spectra of fresh and spent SPC.

| 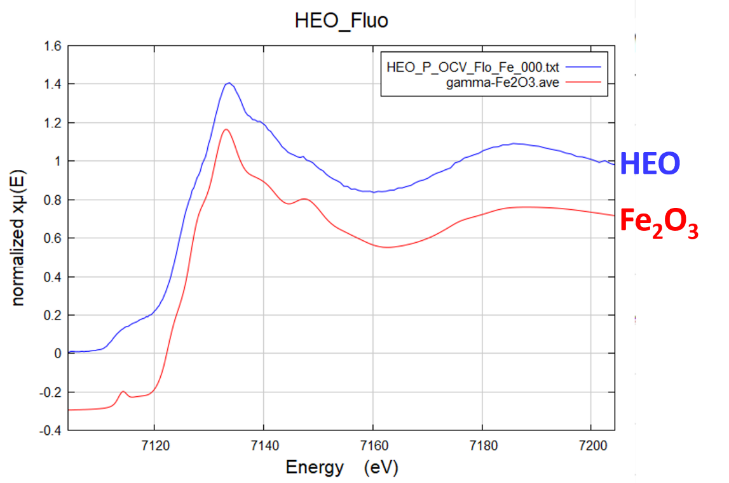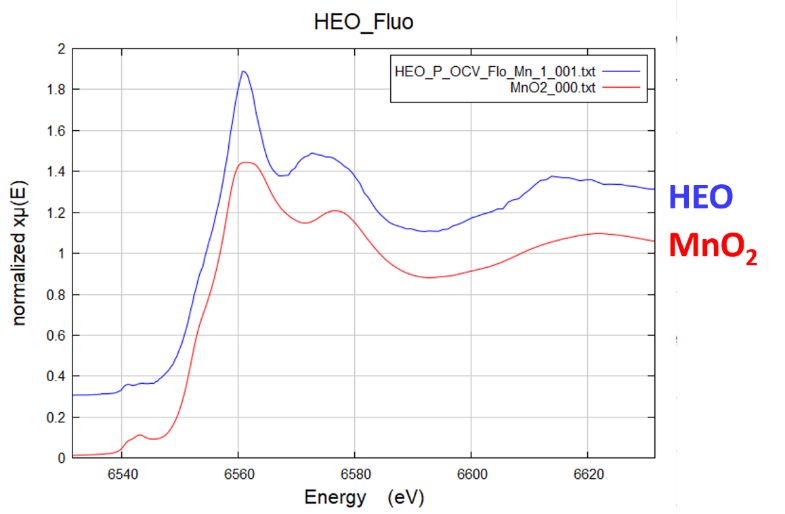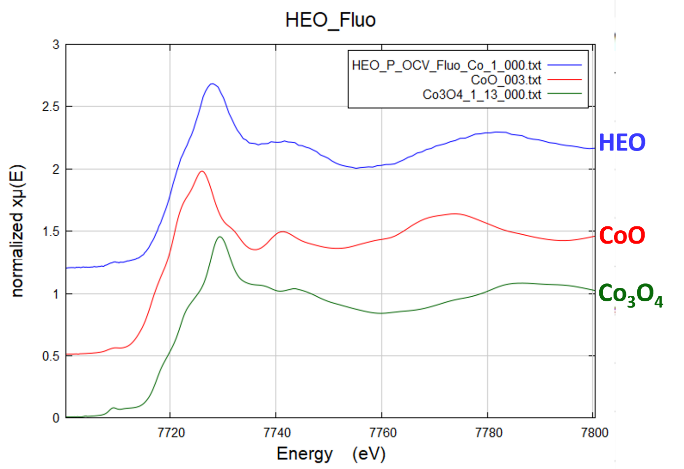 |
| --- |
| **Figure S15**. XANES spectra collected in fluorescence mode at Fe (top), Mn (mid), and Co (bottom) K edges on fresh SPC. Reference spectra are shown for comparison. |


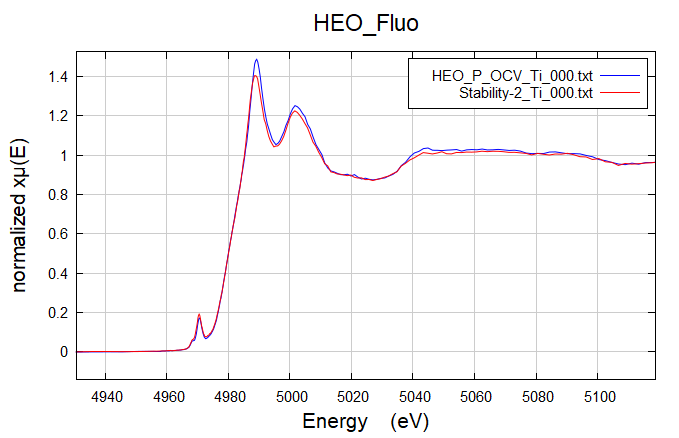

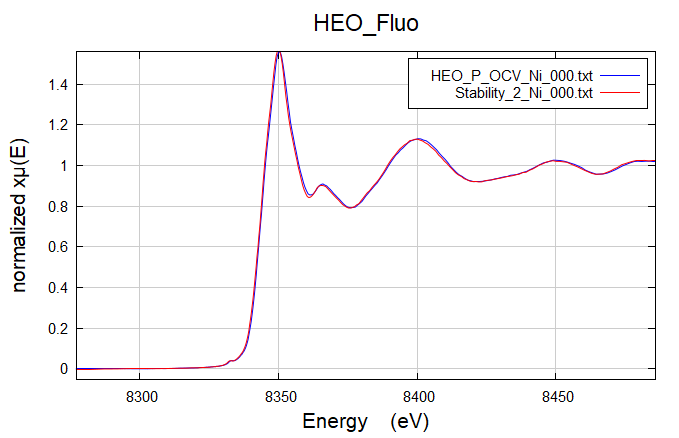

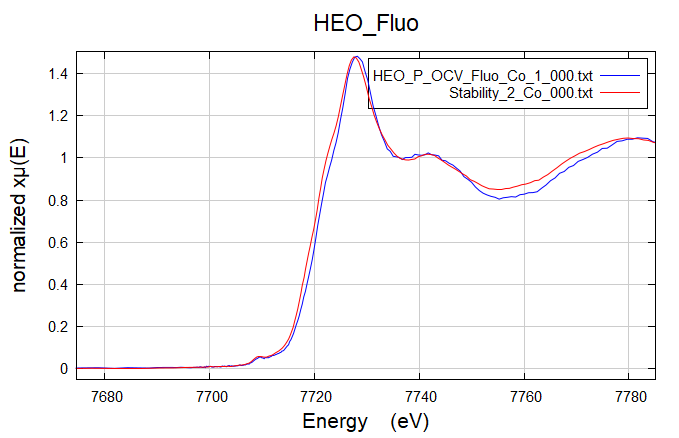

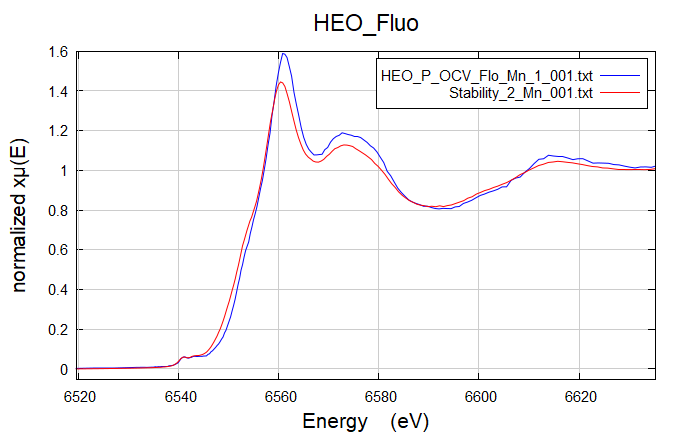

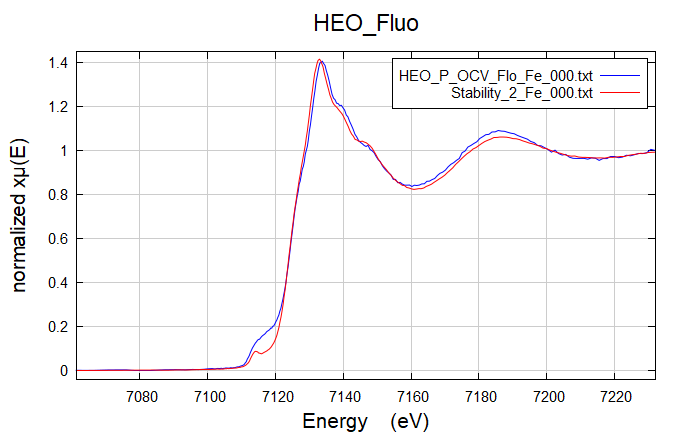


Co

Fe

Ni

Mn

Ti

| **Figure S16**. Comparison of the normalized XANES spectra for fresh (blue) and spent (red) SPC electrodes collected in fluorescence mode at the K edges of Co, Fe, Ni, Mn and Ti. |
| --- |

Figure S17 shows Fourier Transform (FT) of the extracted EXAFS data of the Ni, Ti, Fe, Mn and Co K-edges, collected on the SGC before and after the stability electrochemical tests. Generally speaking, the local structural environment of all elements displays a first and a second atomic shell, due to the TM-O and TM-TM interaction, respectively, but with different level of peaks intensities and/or disordering, even in the fresh electrodes. Some peculiarities can be observed: i) Fe and Ti show broader distribution of interatomic distances compared to the other elements; ii) the Ni displays the same intensity of the first and second peaks, suggesting a regular structure with less structural disorder on the second peak as well. The local environment changes for all elements after cycling, except for Ni. Co shows an important change after cycling, which could be ascribed also to its segregation in the form of spinel. Fe experiences a change in the bond distances rather than in disorder, while Mn shows important disordering and Ti some disordering limited to the first neighbors (shell).

| 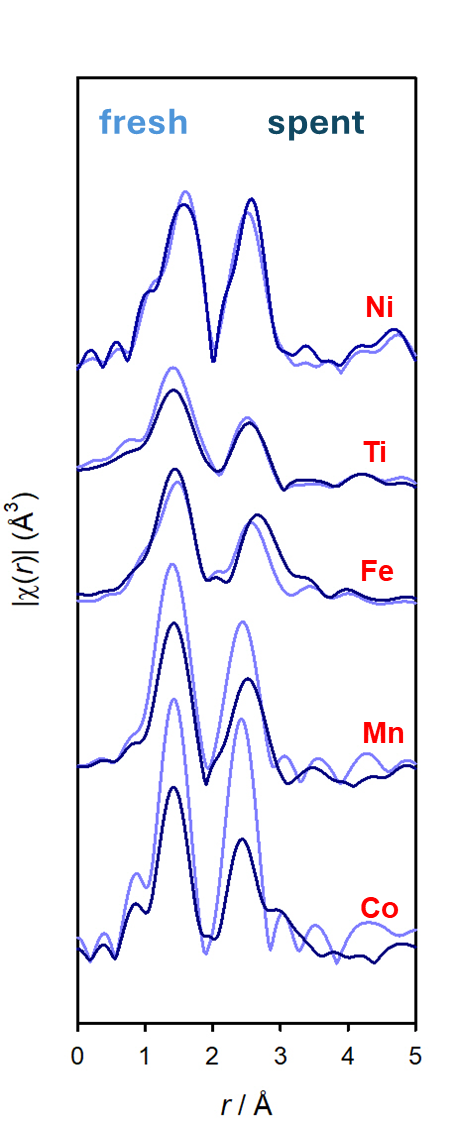 |
| --- |
| **Figure S17.** EXAFS-FT plot of fresh (light blue) and spent (dark blue) SGC. |

**SGC-Na0.9**

In order to compare the effect of a larger content of Na on the overall performance of the SGC, we produced a CAM via the same Pechini sol-gel approach described above, with a composition of Na_0.90_(Ni_0.205_Fe_0.18_Mn_0.205_Co_0.21_Ti_0.20_)O_2_ with the Na determined via ICP and the TM fraction via SEM-EDS. The microstructure is similar to the one observed for the SGC with lower Na content, see Figure S18. The CAM is composed of ~95% layered phases (40.3% O3, 54.4 % P3), the rest is spinel. The XRD pattern, compared to the one with the SG-CAM reported in the main manuscript, is displayed in Figure S19 and the corresponding phase fractions and lattice parameters in Table S8, where they are compared with the SG-CAM described in the main text.

Again, a mixture of O3 and P3 polymorph is observed. The fraction of residual spinel phase is the same in both CAMs. The cell parameters of P3 did not change compared to the SG-CAM with lower Na, while the O3 experienced an important contraction of the *c* axis, which is consistent with a larger fraction of sodium.

| 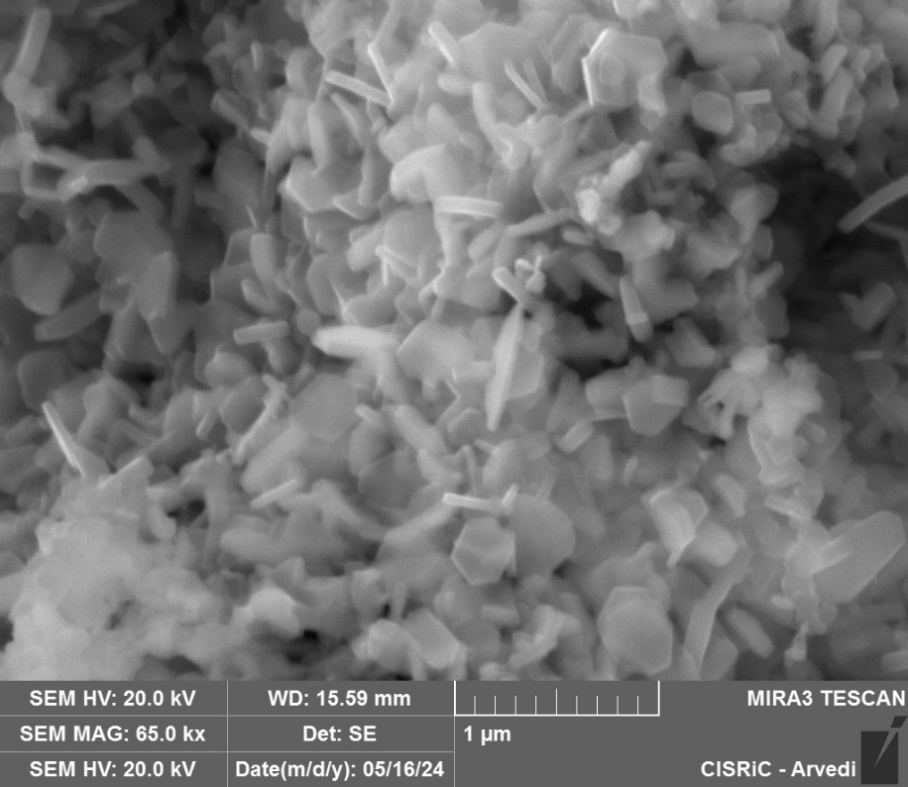 |
| --- |
| **Figure S18**. SEM image with secondary electrons on the SGC-Na0.9 CAM. |

| 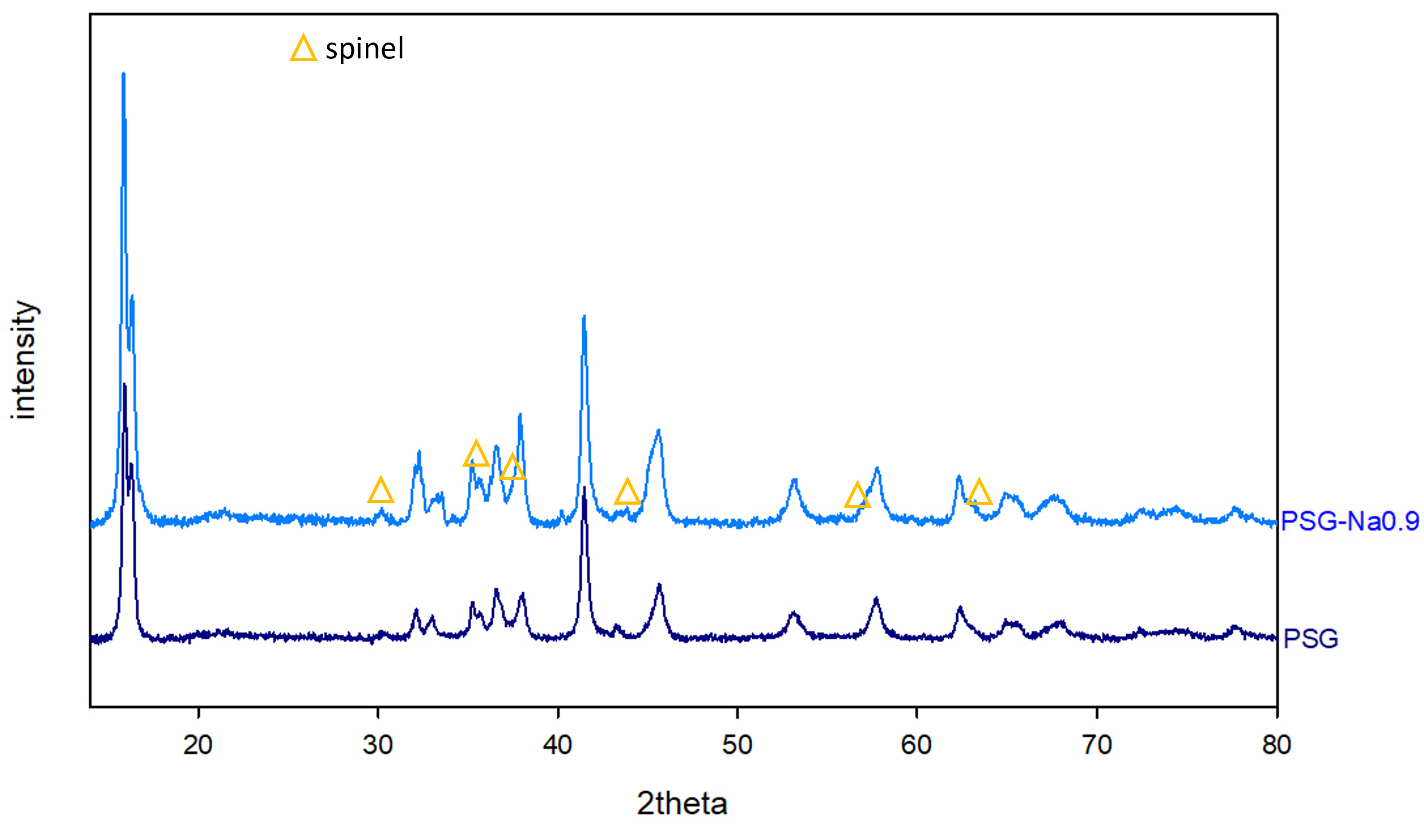 |
| --- |
| **Figure S19.** Experimental XRD patterns of SG-CAMs with different content of Na. |

**Table S8**. Phase fractions and lattice parameters of the SG-Na0.9 CAM determined from Rietveld refinements compared to the CAM with lower amount of Na.

| xNa=0.90 | %wt. | *a* / Å | *c* / Å |
| --- | --- | --- | --- |
| O3 | 40.3 | 2.9742(3) | 16.229(4) |
| P3 | 54.5 | 2.8586(5) | 16.703(5) |
| spinel | 5.3 | 8.346 (3) | - |

| xNa=0.56 | %wt. | *a* / Å | *c* / Å |
| --- | --- | --- | --- |
| O3 | 55.0 | 2.9739(3) | 16.306(3) |
| P3 | 39.8 | 2.8525(3) | 16.703(4) |
| spinel | 5.2 | 8.394(3) | - |

The SG-Na0.9 CAM underwent the same electrochemical testing as the SG-CAM presented in main text. Despite the higher content of Na and the same level of spinel impurity, the specific capacity (both charge and discharge) is much lower than the SG-CAM, as shown in Figure S20.


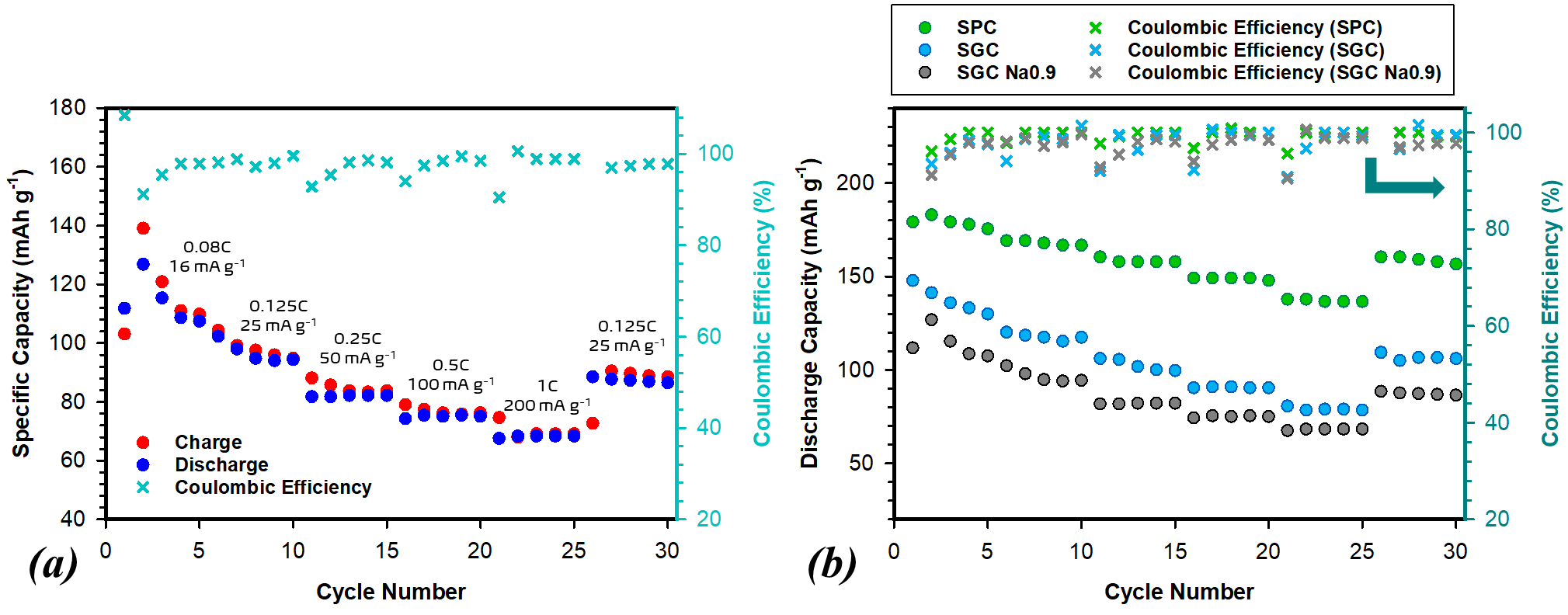


**Figure S20.** (a) Comparison between charge and discharge capacity, coulombic efficiency CE and rate capability test at different current densities for a SGC produced with a Na content of 0.9. (b) Same discharge curves compared to SGC and SPC.

**References**

[1] B. H. Toby, *J Appl Crystallogr* 2001, *34*, 210.

[2] A. C. Ferrari, J. Robertson, *Phys Rev B* 2000, *61*, 14095.

[3] G. Messina, A. Paoletti, S. Santangelo, A. Tagliaferro, A. Tucciarone, *J Appl Phys* 2001, *89*, 1053.

[4] C. Chien, S. Li, W. Lai, Y. Yeh, H. Chen, I. Chen, L. Chen, K. Chen, T. Nemoto, S. Isoda, M. Chen, T. Fujita, G. Eda, H. Yamaguchi, M. Chhowalla, C. Chen, *Angewandte Chemie International Edition* 2012, *51*, 6662.

[5] Y. Luo, Q. Pan, H. Wei, Y. Huang, L. Tang, Z. Wang, C. Yan, J. Mao, K. Dai, Q. Wu, X. Zhang, J. Zheng, *Adv Energy Mater* 2023, *13*.

[6] B. D. Bond, P. W. M. Jacobs, *Journal of the Chemical Society A: Inorganic, Physical, Theoretical* 1966, 1265.

[7] S. Kuboon, Y. H. Hu, *Ind Eng Chem Res* 2011, *50*, 2015.

[8] K. Walczak, A. Plewa, C. Ghica, W. Zając, A. Trenczek-Zając, M. Zając, J. Toboła, J. Molenda, *Energy Storage Mater* 2022, *47*, 500.

[9] M. Nowak, K. Walczak, A. Milewska, J. Płotek, A. Budziak, J. Molenda, *J Alloys Compd* 2023, *968*, 172316.

[10] J. Wang, Q. Li, W. Su, B. Chen, Q. Zhou, X. Wang, *Mater Res Express* 2023, *10*, 125507.

[11] D. A. Anang, J.-H. Park, D. S. Bhange, M. K. Cho, W. Y. Yoon, K. Y. Chung, K.-W. Nam, *Ceram Int* 2019, *45*, 23164.

[12] Y. Pang, Y. Wang, C. Jiang, X. Ding, Y. Xin, Q. Zhou, B. Chen, H. Liu, P. Singh, Q. Wang, H. Gao, *ChemSusChem* 2024, *17*.

[13] J. Yue, F. Xiong, Z. Shadike, X. Gao, J. Chen, L. Pi, Y. Yuan, B. Qu, P. Adamson, L. Ma, Q. Li, P. G. Bruce, *J Power Sources* 2025, *627*, 235735.

[14] H. Deng, L. Liu, Z. Shi, *Mater Lett* 2023, *340*, 134113.

[15] X. Liu, Y. Wan, M. Jia, H. Zhang, W. Xie, H. Hu, X. Yan, X. Zhang, *Energy Storage Mater* 2024, *67*, 103313.
